# Light-based gamma entrainment with novel invisible spectral flicker stimuli

**DOI:** 10.1038/s41598-024-75448-4

**Published:** 2024-11-29

**Authors:** Luna S. Hansen, Marcus H. Carstensen, Mark A. Henney, N. Mai Nguyen, Martin W. Thorning-Schmidt, Jes Broeng, Paul Michael Petersen, Tobias S. Andersen

**Affiliations:** 1 Department of Electrical and Photonics Engineering, Technical University of Denmark, Building 343, Ørsteds Pl., 2800 Kgs. Lyngby, Denmark; 2 OptoCeutics ApS, Nørrebrogade 45C, 4. tv., 2200 Copenhagen N, Denmark; 3 Department of Applied Mathematics and Computer Science, Technical University of Denmark, Richard Petersens Pl., Building 324, 2800 Kgs. Lyngby, Denmark; 4 Centre for Technology Entrepreneurship, Technical University of Denmark, Produktionstorvet, Building 426, 2800 Kgs. Lyngby, Denmark

**Keywords:** GENUS, 40 Hz, Gamma entrainment, Alzheimer’s disease, EEG, SSVEP, Brain network dysfunction, Light therapy, Invisible spectral flicker, Feasibility, Sensory processing, Alzheimer's disease, Inorganic LEDs

## Abstract

Light-based gamma entrainment using sensory stimuli (GENUS) shows considerable potential for the treatment of Alzheimer’s disease (AD) in both animal and human models. While the clinical efficacy of GENUS for AD is paramount, its effectiveness will eventually also rely on the barrier to treatment adherence imposed by the discomfort of gazing at luminance flickering (LF) light. Currently, there have been few attempts to improve the comfort of GENUS. Here we investigate if Invisible spectral flicker (ISF), a novel type of light-based 40 Hz GENUS for which the flicker is almost imperceptible, can be used as a more comfortable option. We found that whereas ISF, LF, and chromatic flicker (CF) all produce a 40 Hz steady-state visually evoked potential (SSVEP), ISF scores significantly better on measures of comfort and perceived flicker. We also demonstrate that, while there is a trend towards a lower SSVEP response, reducing the stimulation brightness has no significant effect on the 40 Hz SSVEP or perceived flicker, though it significantly improves comfort. Finally, there is a slight decrease in the 40 Hz SSVEP response when stimulating with ISF from increasingly peripheral angles. This may ease the discomfort of GENUS treatment by freeing patients from gazing directly at the light.

**Supplementary Information:**

The online version contains supplementary material available at 10.1038/s41598-024-75448-4.

## Introduction

The global prevalence of dementia has more than doubled since 1990^1^, with a new diagnosis occurring every three seconds worldwide^2^. Alzheimer’s disease (AD) accounts for 60 to 80% of dementia cases, making it the most prevalent cause^3^. Given the escalating burden of AD, there is a pressing need for novel disease-modifying therapies^4^. The exact cause of AD is yet unknown. Biomarkers A$\beta$ and tau, established indicators in AD, disrupt axonal transport, leading to cell death and cognitive deficits^4,5^. Neuroinflammation has also been proposed as a mechanism in AD^6–8^, with an extensive immune response contributing to neuronal damage. However, the lack of consensus^9^ on these interactions underscores the complexity of AD pathology.

Despite substantial research in disease-modifying therapies^10^, progress has been limited in the past decade, with notable exceptions such as the recently approved anti-amyloid monoclonal antibodies (MABs) Aducanumab^11^ and Lecanemab. The restricted licensing of Aducanumab in the United States, criticized by many due to efficacy and safety concerns^12–14^, has not been followed by the European Medicines Agency. Lecanemab^15^ shows promising results, exhibiting a significant reduction in amyloid markers and less cognitive decline in the active group compared to placebo. While anti-amyloid MABs therapy has shown positive effects on amyloid load, it is associated with side effects^16^, including amyloid-related imaging abnormalities (ARIA) and infusion reactions, and its clinical impact remains limited^17^. Therefore, further exploration of non-pharmacological interventions beyond amyloid-focused approaches^18,19^, including novel approaches for neurodegenerative disorders^16^ remains imperative.

Recently, alternative approaches such as Electrophysiological alterations have shown promising insight into the neuropathological aspects of AD^20^. AD is among a group of neurological and psychiatric disorders in which a decrease in the power of gamma (40 Hz) oscillations and brain network dysfunction (BND)^21,22^ are observed. Despite this decrease in gamma, studies of the electroencephalography (EEG) spectrum in AD have focused mostly on the low-frequency bands ($<35$ Hz)^22–27^ and have shown a general tendency toward slowing of oscillatory activity primarily with a decrease in the alpha band. Alpha-band and gamma-band activity^28,29^ both play an active role in information processing, with alpha-band activity serving dual functions in inhibition and timing of attention^30^. While spectral changes also occur in the normal aging brain, including slowing of activity mainly by a decrease in alpha power and frequency^31^, this is more prominent in AD^32–34^. Evidence even suggests a correlation between the slowing of the oscillatory activity and the progression of AD exists^23,35^. The exact link between gamma activity and AD is challenging to establish due to the high inter-subject variability in gamma activity that is observed even among healthy subjects^36^. Furthermore, contradicting findings of the resting state EEG in patients with AD report both reduced^36^ and increased^37^ gamma activity, the latter being explained by a compensatory increase in mental exertion by the AD group. The interneurons^38,39^ may have a key role for the electrophysiological changes and cognitive symptoms that occur with AD. Particularly parvalbumin-positive interneurons^40,41^, which are responsible for cortical communication and play a role for cognitive behavior^42^. Especially 40 Hz activity resonates with the inhibitory interneurons and generates oscillatory rhythms during higher brain functions like attention^43^. It is unknown whether the decrease in gamma power in AD patients is a cause or an effect. This leads to the hypothesis that the progression of AD might be slowed if gamma power could be increased.

New evidence has shown that activation of gamma activity using 40 Hz stimulation has great potential for the treatment of AD with clear positive effects in mouse models^44–47^. Stimulation of AD mouse models with 40 Hz light-based stimulation resulted in decreased A$\beta$ and tau load, an increase in microglia recruitment, and additional neuroprotective effects^46–48^. While clinical benefits are not yet certain, 40 Hz can be induced in human participants as measured with EEG and has shown promising therapeutic potential^49–52^. Recent independent studies on patients with AD showed a significant reduction in white matter atrophy and a decrease in cognitive decline after administering 40 Hz light stimulation one hour daily^51,53,54^. Inducing 40 Hz activity in the brain using transcranial magnetic stimulation has also been shown to improve cognitive function for up to 8 weeks after treatment in patients with probable AD^55^. Gamma stimulation therapy might have considerably fewer side effects than pharmacological interventions^56,57^, and may be a supplement to pharmacological therapy. Various 40 Hz stimulation methods exist, such as sensory stimulation (tactile^58^, visual^51,59^, auditory^60^, or a combination^61^), transcranial magnetic stimulation (TMS)^55^, transcranial alternating current stimulation (tACS)^62,63^, and Transcranial photobiomodulation (tPBM)^64^. Each method has distinct advantages and disadvantages impacting both efficacy and usability. These include the power of the evoked response but also the user experience in terms of tolerability, safety, and possible side effects.

The term GENUS (Gamma Entrainment Using Sensory Stimuli) has been adopted to describe stimulation involving sensory inputs. Most studied is the visual and auditory, or a combination of the two. Especially, 40 Hz audiovisual stimulus has been used in mice trials^65^ and clinical trials^52,57,61^ as multi-sensory stimulation has the potential to reach wide brain regions^66^. Studies have found that audiovisual 40 Hz stimulation can reach subcortical structures beyond the cortex including the hippocampus^61^, better than visual or auditory stimulation alone^67^. However, visual 40 Hz stimulus alone has also demonstrated the ability to propagate to the hippocampus, particularly in combination with a cognitive task^68^. Due to its non-invasiveness, stimulation using the visual pathway alone can have greater potential for success in terms of feasibility and real-life application.

Light-based stimulations are generally considered safe, primarily due to their non-invasive nature involving the application of light. However, visual stimulation includes *flickering* light, hence lies a risk. Exposure to flickering light has mild risks which may include eye strain and fatigue, while severe risks include migraines and seizures in patients with photosensitivity. Currently, no studies have reported any severe adverse events related to the use of GENUS treatment^57,61^. Guidelines from authorities like the IEEE and CIE should always be considered in the design of visual stimulation devices. For commercially sold LED appliances, the current IEEE guideline recommends a maximum 5% modulation for flicker frequencies below 90 Hz^77,78^.

Visual 40 Hz stimulation uses temporal light modulation (TLM), modulating a light source at 40 Hz. The light can be modulated in either luminance (i.e. brightness) resulting in luminance flicker (LF) or chromaticity (i.e. color composition) resulting in chromatic flicker (CF) or a combination of the two. White luminance flicker and red-green chromatic flicker have been found to evoke the highest cortical response^69–76^.

The perception and sensitivity to visual flicker are highly subjective and vary depending on the stimulus. The critical flicker-fusion frequency (CFF)^79^ is the threshold frequency above which flicker is not perceived. For luminance flicker with a 100% modulation depth, the CFF threshold lies in the range of 60-100 Hz^78,80^. In patients with AD, the CFF is found to be significantly lower^81^. For chromatic flicker, the CFF depends on the colors used (as well as brightness, field-of-view, and waveform) but lies in the range of 25–50 Hz^82–86^. Studies have shown that CF light can induce a cortical response with a minimum sensation of flicker^87^ and that a neural response can be measured with flicker stimulus even beyond the CFF^88,89^. Therefore, CF may be a more appropriate implementation of visual 40 Hz stimulation to improve comfort.

According to the IEEE, *flicker* is defined as modulated light that is perceived as flickering^78^. The IEEE^78^ furthermore defines *Invisible flicker* as light where the modulation can be sensed but not perceived. Invisible Spectral Flicker (ISF) is a novel technique to mitigate the perception and minimize the sensation of flicker^90^. ISF alternates between two phases of white light that are of similar color temperature but spectrally different. Stimulation with 40 Hz ISF also evokes a strong 40 Hz cortical response^91^.

In previous pre-clinical and clinical literature, there has been a high degree of focus on the ability to evoke a (strong) cortical 40 Hz response rather than the user experience associated with the treatment implementation. A recent study on the safety and feasibility of GENUS with combined 40 Hz flicker and audiostimulation 1-hour daily for 6 months (n = 74 included) resulted in a 28.38% dropout rate^92^. These results highlight the importance of treatment feasibility to reduce the dropout rate, increase the duration of correct usage, and ultimately optimize efficacy and effectiveness. In comparison, a similar study using 1-hour daily visual stimulation for 6 weeks (n = 11 included) reported no dropouts: Although, the study had a shorter duration and a smaller sample size^51^. Importantly, the treatments were implemented differently in the two studies. Hajós et al.^92^ used wearable glasses and earpieces, whereas Agger et al.^51^ used a standalone table-top light stimulation device. The designs of the treatment may play a vital role in participant retention and adherence as interventions demanding active engagement may face neglect. Adherence is key to any treatment and reliable measures are needed to truly evaluate the efficacy of a treatment.

**Fig. 1:**
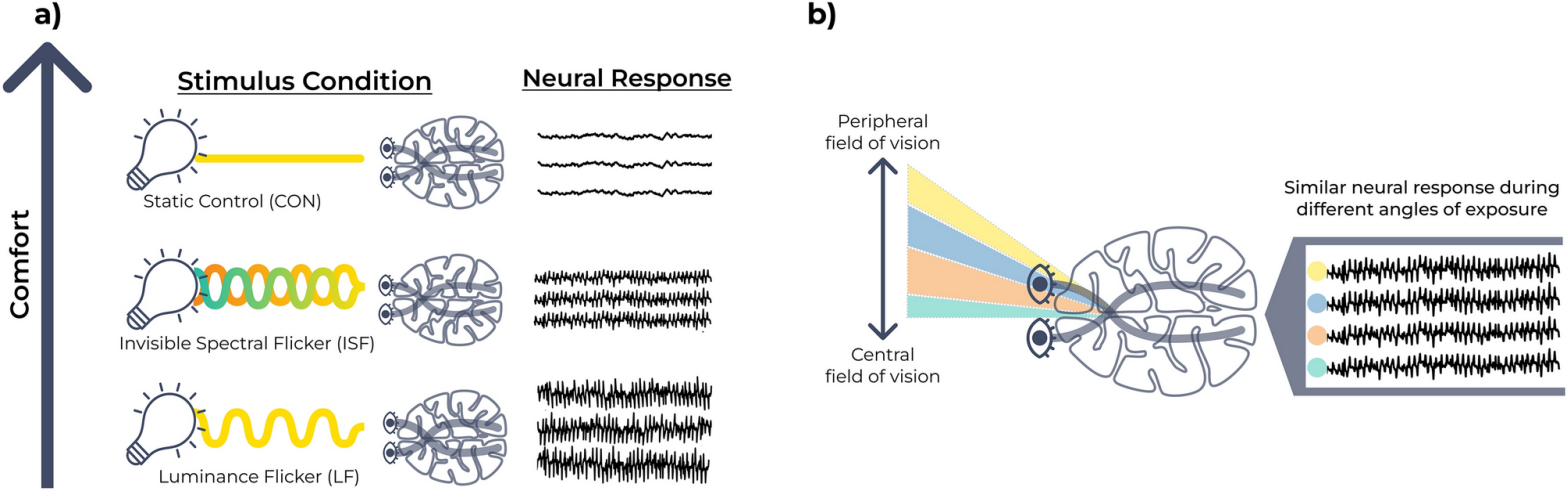
Graphical illustration of the neural response during 40 Hz visual stimulation. (**a**) Administration of exogenous 40 Hz temporally modulated light evokes an electrical neural response oscillating at the same frequency as the flickering light stimulation. ISF evokes a lower response than LF but is rated more comfortable. (**b**) ISF will evoke a response from direct exposure but also by stimulating at a peripheral angle.

In this study, we aim to understand how LF, CF, and ISF impact gamma entrainment and user experience (see Fig. 1). The study contains three experiments. In experiment A, we measure the steady-state visual evoked potential (SSVEP) response of ISF, LF, and CF at different levels of brightness to investigate the effects of brightness and stimulus. In experiment C we investigate how the brightness and stimulus affect the perceived flicker and experienced discomfort. In experiment B we measure the SSVEP response from ISF to compare the effect of stimulating the peripheral, rather than the central, field of vision.

We hypothesize that ISF, while inducing a lower acute response^91^, has a lower level of perceived flicker and results in higher comfort compared to LF and CF, thereby, increasing its potential for real-world application. We propose that better visual stimulation options can be achieved by rendering the flicker experience from TLM near imperceptible and freeing the user from active involvement.

## Methods

### Participants and ethics

Twenty-five healthy participants between the ages of 20 to 44 (15 males and 10 females) without vision impairment were recruited through the Technical University of Denmark (DTU) (Anker Engelunds Vej 1, Bygning 101A, 2800 Kongens Lyngby, Denmark). Participants were selected based on exclusion criteria, excluding those above the age of 50 and individuals with a history of light sensitivity, neurological or psychiatric diseases, epilepsy, or familial history of epilepsy. Participants underwent a self-reported assessment guided by questions from the investigator. This approach identified individuals with potential photosensitivity or known neurological or psychiatric disorders. One participant was excluded based on their self-reported photosensitivity.

All participants completed every experiment, but due to recording issues and missing data, only a subset of 20 participants (7 females) could be used in the analysis (see further details in supplementary materials). Before inclusion, written and verbal information was provided to the participants, and written informed consent was obtained. The recruitment and experimental procedures adhered to the Declaration of Helsinki, the General Data Protection Regulation (GDPR), and were approved by the Institutional Review Board at DTU Compute, Technical University of Denmark (application number: COMP-IRB-2020-01, approved 14-02-2020).

### Visual stimulation system

During all three experiments (A, B and C) the Visual Stimulation System (VSS1.0) developed by OptoCeutics ApS was used. The device includes a flat light diffuser screen of width 25 cm and height 18 cm (see Fig. 3b). The device is equipped with two sets of six LEDs (blue, cyan, green, lime, amber, red) that can be customized for various light settings. The LEDs are a mix of direct and phosphor-converted emitters. A specialized LED controller is employed to facilitate precise adjustment of the brightness for each of the six independent channels. This ensures precise calibration of the 40 Hz stimulation.

A total of four different types of light were designed for the experimental stimuli. A modified version of the VSS device using only the blue, red, green, lime, and red LEDs was used for the experiment. Each LED channel was independently controlled, to produce the different types of light output needed. The visual stimuli included a 40 Hz luminance flicker (LF), a 40 Hz invisible spectral flicker stimulus (ISF), a 40 Hz color-fused red/green stimulus (CF), and a non-flickering control light (CON). Modulation depth of 100%, 5%, 25%, and 0% respectively. The stimulation frequency was calibrated to 40 Hz and manually measured using an oscilloscope before and after the data collection to 40.01 Hz, corresponding to an acceptable 0.25% deviation. All the 40 Hz modulated stimuli had a 50% duty cycle. Except for the color-fused stimulus, the light-based stimuli were designed to have similar white appearance and spectral composition (see Fig. 3d and Supplementary Material Fig. S3). The color temperature of the LF, CON and ISF lights were matched at 2572 K and CF at 1717 K. In experiments, A and C, the brightness of the four types of light varied in intensity by three levels - approximately 5,200, 9,100, and 10,400 lux - measured at the surface of the device. This corresponds to luminance values of 1577 ${\mathrm{cd/m^2}},$ 2589 ${\mathrm{cd/m^2}}$ and 3019 ${\mathrm{cd/m^2}}$. Stimuli were selected based on existing literature for entrainment from chromatic and luminance flicker stimuli^69–76^. We addressed a gap in the literature by comparing brightness, discomfort, and flicker perception. Following prior and ongoing studies (NCT04574921, NCT05260177), we selected the lowest brightness and explored higher levels at 75% and 100% increases.

To ensure a similar experience of brightness between the four types of stimuli, the brightness was visually matched. The total lux value was not just integrated over a full cycle (Supplementary Material Fig. S2). Instead, the energy was preserved over half a cycle (12.5 ms) as both the SSVEP measure of interest and the brightness are perceived on a millisecond level. For experiment B, only a single light stimulus (ISF) was used at the highest level of brightness. Marked fixation points were used to help the participants focus^76^, and participants were carefully instructed before each experiment on where to look.

### Experimental paradigm

The study included three experiments; two successive EEG experiments (A and B) followed by a separate assessment of the different lights (experiment C). Experiments A and C measured the EEG response and subjective discomfort rating for four types of light stimuli (LF, CON, ISF, CF) at three different levels of brightness. Experiment B measured the EEG response for ISF under varying exposure angles. The three experiments are considered independent and participants were reminded about the conditions before each experiment. The data were collected during a single experimental visit and the total time of the experiment visit was no longer than 90 minutes.

#### Experiment A

A within-subject design was used with four types of light stimuli and three light intensities, crossed to obtain a total of 12 stimuli. Each of these was repeated six times. The order of the 72 stimuli presentations was randomized individually for each test participant. The order was validated using graphical methods post hoc. Repetitions in random order were incorporated into the experimental design and analysis to account for potential effects arising from the perceiver’s adaptation to the light stimulus, including aspects such as attention fluctuations and neural adaptation. Each stimulus was presented for 20 seconds with an interstimulus interval (ISI) of 5 seconds. Before and after the sequence of stimuli, a 60 second eyes-open baseline was recorded. The total experiment duration was 32 minutes. The participants were asked to rest their gaze at a fixation point at the center of the light source (Fig. 3b).

#### Experiment B

**Fig. 2:**
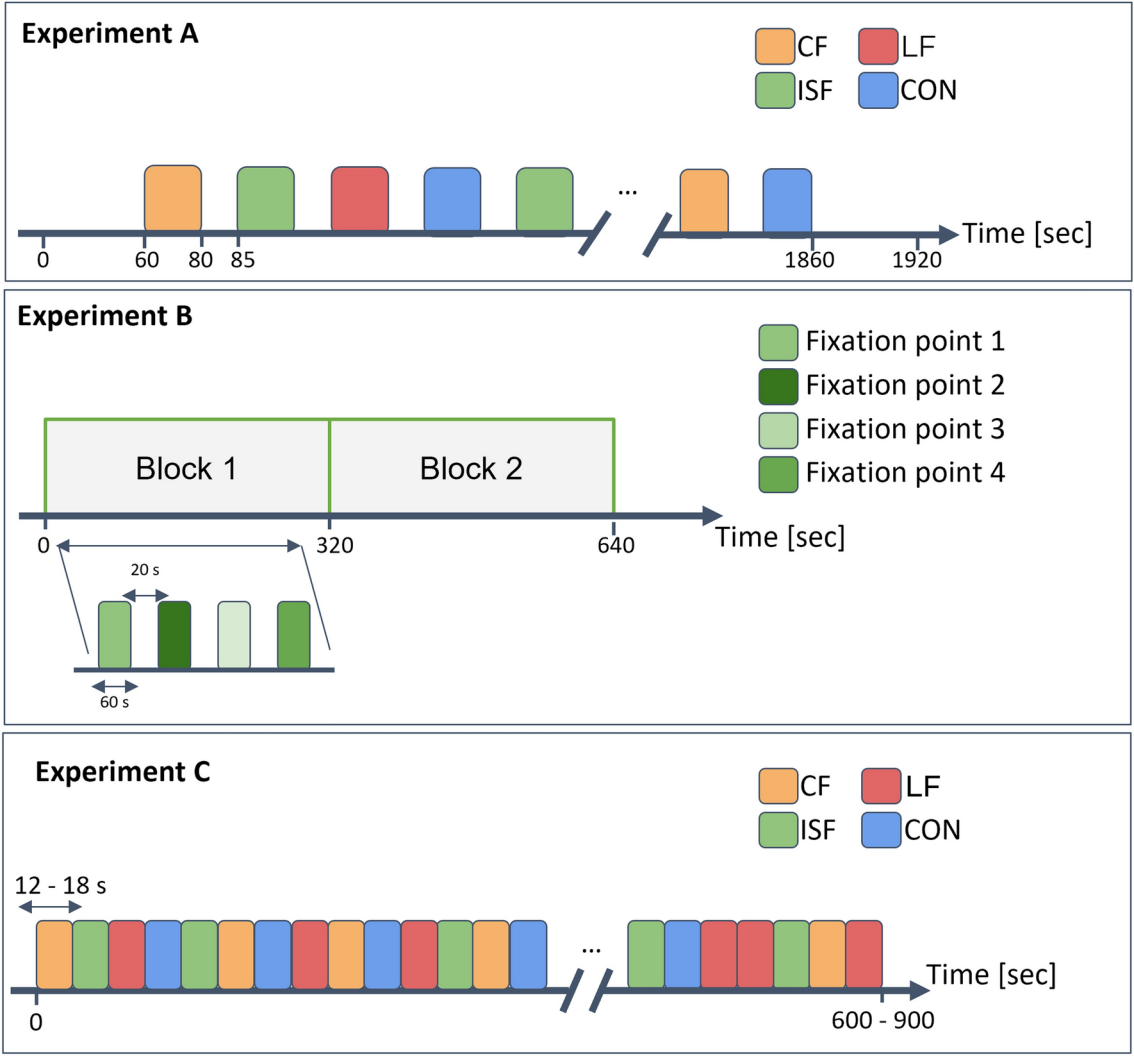
Experimental Designs: Experiment A (top) included four different types of light stimulus (ISF, CF, LF, and CON) at three levels of brightness. (Brightness is not illustrated in the figure). Each stimulus was presented six times and the order was randomized. An interstimulus interval (ISI) of five seconds was introduced between each stimulus. 60 seconds baseline with eyes-open and no light was also recorded at the beginning and the end of the experiment. EEG was recorded during the experiment. Experiment B (middle) included light stimulation with ISF. Participants were asked to gaze at different fixation points only once in each of the two blocks. The order within each block was random. The light stimulus was turned on for 60 seconds, followed by 20 second ISI to allow for verbal instructions on the next fixation point. EEG was recorded during the experiment. Experiment C (bottom) included the same 12 light stimuli as experiment A, however, participants would rate each of the stimuli in terms of perceived discomfort and flicker. Each stimulus was presented four times in random order. The stimulus would change when rating was completed, and pace of the experiment was thereby controlled by the participant.

**Fig. 3:**
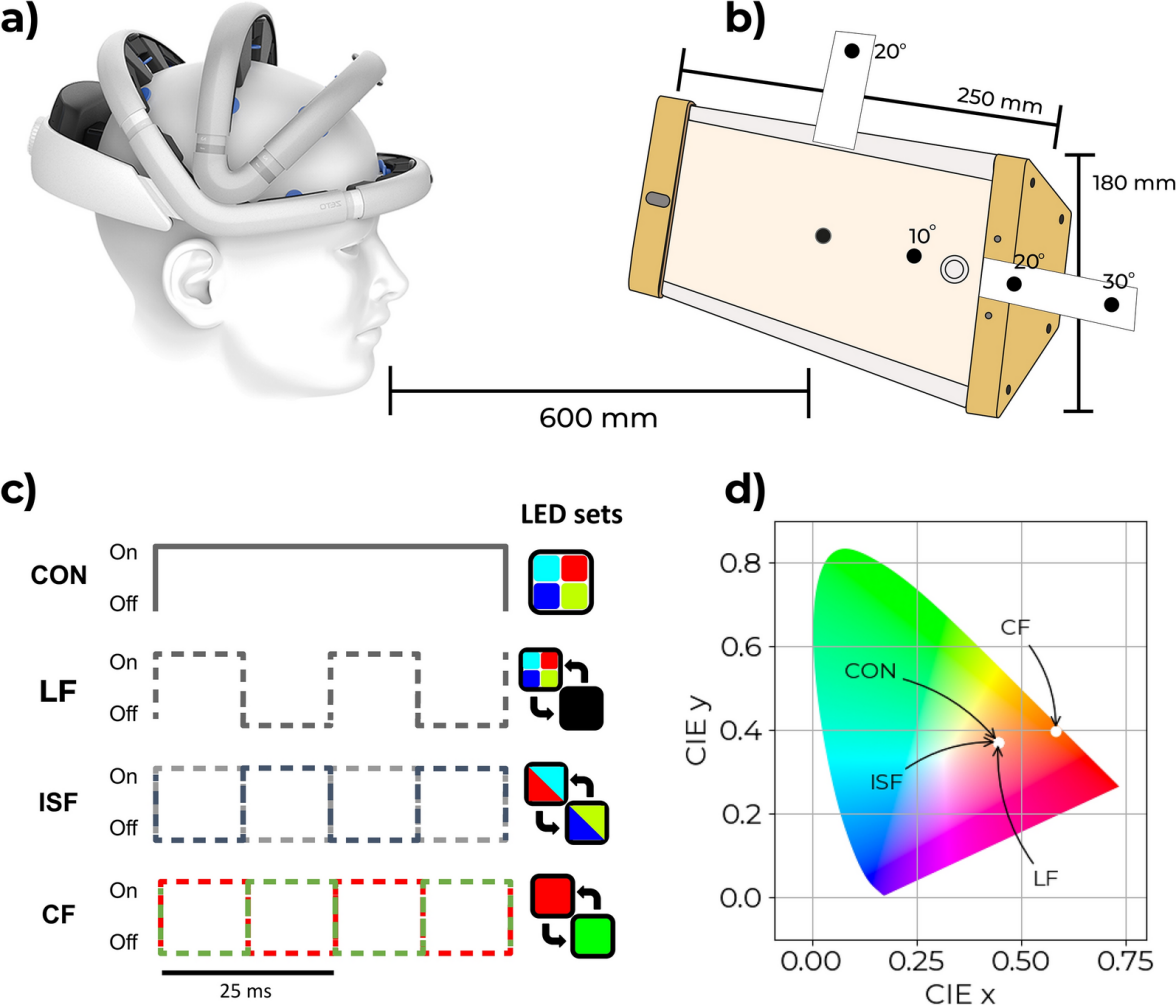
Experimental setup. Participants were placed approximately 60 cm from the light device and instructed to rest their gaze at different fixation points according to the experiment. During experiment A, participants were instructed to gaze at the center fixation point (indicated with no angles). During experiment B, participants were instructed during the ISI about which fixation point to gaze at during the following stimulation period. Four light settings at three different levels of brightness were used in experiment A; a non-flickering control light (CON), a 40 Hz luminance flickering light, a 40 Hz invisible spectral flicker (ISF) light, and a 40 Hz red/green color-fused flickering light (CF). Light settings are designed as given in (**c**) and (**d**). LF, CON, and ISF are all positioned at the same coordinates (0.41, 0.34) in the CIE diagram and therefore appear visually similar to the observer. CF does not have a similar appearance and is therefore positioned at different coordinates (0.58, 0.40). The coordinates of the two half-cycles of ISF are (0.52, 0.36) and (0.3, 0.32).

A within-subject study design with two blocks was used. Four different fixation points were used for each subject and repeated only once in each of the two blocks. The order within each block was random (Fig. 2). Like experiment A, repetitions were included to account for attention and adaption. The order of gazing points was randomized for each individual before the experiment for the investigator to instruct the participant to move their gaze. The order was validated before the experiment and post hoc. During this experiment, 40 Hz ISF was presented for 60 seconds, and during a 20 second ISI, the participant was instructed to look at a new fixation point placed on or around the stimulation device. These included two fixation points within the perimeter of the device; at the center and 10° to the right, and three fixation points outside this perimeter; at angles 20° to the right, 30° to the right, and 20° above, see Fig. 3a, b. The total experiment duration was 10 minutes. Participants were stratified into two groups. The first 10 participants were instructed to gaze at points along the horizontal angles, while the remaining 10 participants, were instructed to gaze at fixation points outside of the device. The center gaze was included in both groups as a reference.

#### Experiment C

A within-subject design was used with four repetitions of each stimulus and complete randomization of stimulus order. In the experiment, participants were exposed to the same 12 lights as experiment A while simultaneously rating their experienced discomfort and perceived flicker using a PC as described in the following section. The duration of the experiment was 10 to 15 minutes but varied according to each participant. The same randomization as in experiment A was applied.

### Electroencephalography recording

All experiments were carried out in a purposely designed EEG laboratory with semi-blinded windows. Electrical interference was minimized by disconnecting all non-essential electronic devices and ambient light. The stimulus system, VSS 1.0, being the only powered device, was supplied using a shielded cable to minimize noise. To further reduce noise and artifacts from the participants, they were seated in a comfortable chair approximately 60 cm from the stimulus device. Participants were instructed on the importance of remaining still and relaxing throughout the EEG recording.

EEG data was collected using a FDA-approved wireless 19-channel headset (Zeto Inc. medical device manufacturer, Santa Clara, CA.). The system uses dry active electrodes, it has a bandwidth of 0.003– 250 Hz and noise levels are $<1\,\upmu{\text{V}}$ RMS. Electrodes were placed according to the international 10-20 system and the following 12 channels were used for data analysis: Fp1, Fp2, Fz, F3, F4, T5, T6, P3, P4, Pz, O1 and O2. Fpz was used as ground and linked mastoids as reference. Data was sampled using dedicated software at a sampling rate of 500 Hz via Bluetooth connection.

### Subjective light assessment

An assessment scale was adapted from the visual analogue scale (VAS), including the following three measures: 1) Discomfort (“How much discomfort do you experience?”), 2) flicker (“How much flicker do you experience?”), and 3) color (“How do you like the color?”). Participants were instructed to evaluate each stimulation based on the three specified assessments using an 11-point scale ranging from zero to ten. Ten being maximum discomfort, maximum flicker, and very pleasant, respectively. The assessments were conducted utilizing a PC, with the paradigm programmed in Python. Participants had unsupervised control over the pace of the assessment, initiating each evaluation by interacting with the program. The resulting data were stored in a CSV format. It is noteworthy that the light remained illuminated during the assessment and underwent changes upon interaction. By instructing participants to independently rate various factors, we employ the method of controlled comparisons to ensure an isolated assessment of discomfort irrespective of color and flicker influences. Controlled comparison facilitates a more transparent and interpretable analysis of the direct correlation between comfort ratings and underscores the immediate influence of comfort ratings on the overall lighting experience. Supplementary materials include further elaboration of the experimental setup.

### EEG data pre-processing

At the conclusion of the experiments, EEG data was processed with Python using the MNE toolbox (version 0.24.1)^93^. Data was filtered using a zero-phase finite impulse response (FIR) notch filter at 50 Hz using a hamming window and transition band of 0.5 Hz, followed by a 1 Hz high pass filter of the same type but with a transition bandwidth of 1 Hz. Data was manually inspected to reject bad channels and mark bad segments of data. One recording from experiment A and one recording from experiment B were excluded completely based on the manual inspection of the data, due to inadequate signal quality from excessive movement and insufficient electrode connection. The process of manual inspection involved a visual examination of the data to identify and reject bad channels and mark segments unsuitable for analysis. While no specific predefined criteria or thresholds were utilized, the decision was based on a careful visual assessment of the data quality. Re-referencing to the common average was also applied along with Independent component analysis (ICA) to remove ocular artifacts. ICA was applied using the Picard algorithm to remove ocular artifacts. Ocular artifacts were discovered by visual inspection of the raw components along with their source location. The number of components that contain ocular artifacts varied depending on the recording. However, in general, we excluded one or two components dominated by ocular artifacts. Eye movements and blinks generate strong, non-Gaussian signals that can easily be separated from the brain activity by ICA. In a few cases, the ocular artifacts were particularly strong and we found more than two ICs.

### Response quantification

It is challenging to quantify EEG spectral power and SSVEP peaks as the signals are inherently noisy due to various sources, such as muscle activity, eye movements, and electrical interference with a high subject variability. Multiple methods have been proposed in the literature including, absolute power, relative power, and baseline comparisons. The study aims to assess how 40 Hz stimulation enhances brain activity. Thus, we require a method to quantify the peak relative to the baseline. However, due to the experiment’s design and duration, obtaining a valid baseline recording was deemed impractical. This is mainly because of potential temporal fluctuations caused by motion and changes in electrode impedance thereof. The following method of estimating the SNR was chosen for its simplicity and available documentation^94,95^. However, the method exhibits high sensitivity to noise, and alternative approaches, such as contrast-to-noise ratio or more sophisticated techniques like FoooF (fitting oscillations and one over f), may offer improved accuracy by considering the variance in noise.

The power spectral density (PSD) was estimated by the Welch method with a Hann window of 10 seconds and 50% overlap. Segments were zero-padded to obtain a frequency resolution of 0.05 Hz. For the best estimation of the 40 Hz SSVEP peak, the frequency resolution was chosen as an integer multiple of the flicker cycle. The Welch method for PSD estimation was chosen due to its widespread use and established balance between frequency resolution and variance reduction. The Hann window was chosen for its smooth, bell-shaped shape that tapers off gradually at the edges, reducing spectral leakage. The larger window size and high overlap percentage blur the temporal resolution. However, it reduces noise or transient fluctuations in the signal which is favorable for our analysis. The selection of frequency resolution was based on a balance between capturing all details in the spectral domain and minimizing computational demands. The frequency resolution of 0.05 Hz was achieved through zero-padding. Opting for a lower frequency resolution was avoided to prevent excessive smoothing, which could result in the loss of finer structure in the spectrum. This resolution was deemed adequate based on the characteristics of the SSVEP under investigation.

For each frequency bin in the power spectrum, the signal-to-noise ratio (SNR) was computed relative to the average of the nearest two adjacent integer frequencies skipping the nearest integer frequency on both sides, corresponding to a frequency band from $\pm 1$ Hz to $\pm 2$ Hz around each target frequency. The averaging was computed by convolving with a square kernel,^94,95^. The SNR in the frequency bin closest to 40 Hz was extracted, averaged over channels and used in the statistical analysis.

### Statistical analysis

To analyze the SNR of the SSVEP, a linear mixed effect model (a within-subject design) with a significance level of 0.05 was used. Participants and repetitions were included as random effects, whereas stimulus conditions (type of light, brightness, or exposure angle) were included as fixed effect(s). In experiment B, block was added as a random factor. Interaction effects between fixed and random effects were included. With this model it is assumed that there is a linear relationship between the independent and dependent variables and both residuals and random factors are independent, normally distributed, and homoscedastic. These assumptions are checked by Q-Q plots and scatter plots of the fitted- versus residual values to determine that there is no residual correlation structure unaccounted for. The discomfort measures were analyzed using a cumulative mixed effect^96^ model due to the ordinal nature of the Likert-item type data. The model included the type of light and brightness as fixed effects along with the subject as random effects. Model reductions were applied and the final models are presented here. The cumulative model accommodates the nested structure of repeated measurements within participants, and its flexibility in incorporating fixed and random effects is advantageous for capturing both population-level trends and individual variability. However, it’s crucial to note that the model assumes proportional odds and that the outcomes are less straightforward to interpret. The proportional odds assumption states that the effect of the independent variables on the odds of moving from one rating category to the next is the same across all levels of rating. The assumption was tested using graphical methods by visually inspecting the estimated distance between the odds of each rating score.

The final models, where the dependent variable, *Y*, is either the SNR values, experienced discomfort, or flicker, are expressed in Eqs. (1–3). For experiment A:

1 $$\begin{aligned} Y_i =&\mu + \alpha ({\text{stimulus}}_i) + \beta ({\text{brightness}}_i) + c({\text{repetition}}_i) + d({\text{subject}}_i) \\ &+ m({\text{stimulus}}_i, {\text{brightness}}_i, {\text{subject}}_i) + \epsilon _i,\end{aligned}$$

where $$Y_i$$ is the 40 Hz SNR for the *i*th trial averaged across electrodes, $$c\sim N(0, \sigma _{{\text{repetition}}}^{2}),$$$$d \sim N(0,\sigma _{{\text{subject}}}^{2}),$$$$m\sim N(0,\sigma _{{\text{stimulus,brightness,subject}}}^{2}),$$ and $$\epsilon \sim N(0,\sigma ^2).$$

For experiment B:

2 $$\begin{aligned} Y_i = \mu + \alpha ({\text{angle}}_i) + d({\text{subject}}_i) + g({\text{angle}}_i, {\text{subject}}_i) + \epsilon _i,\end{aligned}$$

where $Y_i$ is the 40 Hz SNR for the *i*th trial averaged across electrodes, $g\sim N(0, \sigma _{{\text{angle, subject}}}^{2}),$ and $\epsilon \sim N(0,\sigma ^2).$

For experiment C with both outcome measures:

3 $$\begin{aligned} Y_i = \mu +\alpha ({\text{stimulus}}_i) + \beta ({\text{brightness}}_i) + d({\text{subject}}_i) + \epsilon _i,\end{aligned}$$

where $Y_i$ is the outcome for the *i*th trial, $d\sim N(0,\sigma _{{\text{subject}}}^{2}),$ and $\epsilon \sim N(0,\sigma ^2 ).$

Post-hoc comparisons with Šidák corrections for multiple comparisons were applied to compare the different stimulus conditions. All statistical analyses were done in R. Specifically, the validated functions *lmer* (lmerTest/lme4)^97^ and *ordinal* (clmm)^98^ were used for estimating the mixed effect models. The *emmeans* package was used for correction of multiple comparisons. Additionally, Bayesian ANOVA was applied, using JASP, to the mean values estimated by the models to compare the peripheral angle conditions in experiment B with the control stimulation in experiment A.

## Results

### Experiment A: stimulus type and brightness

**Fig. 4:**
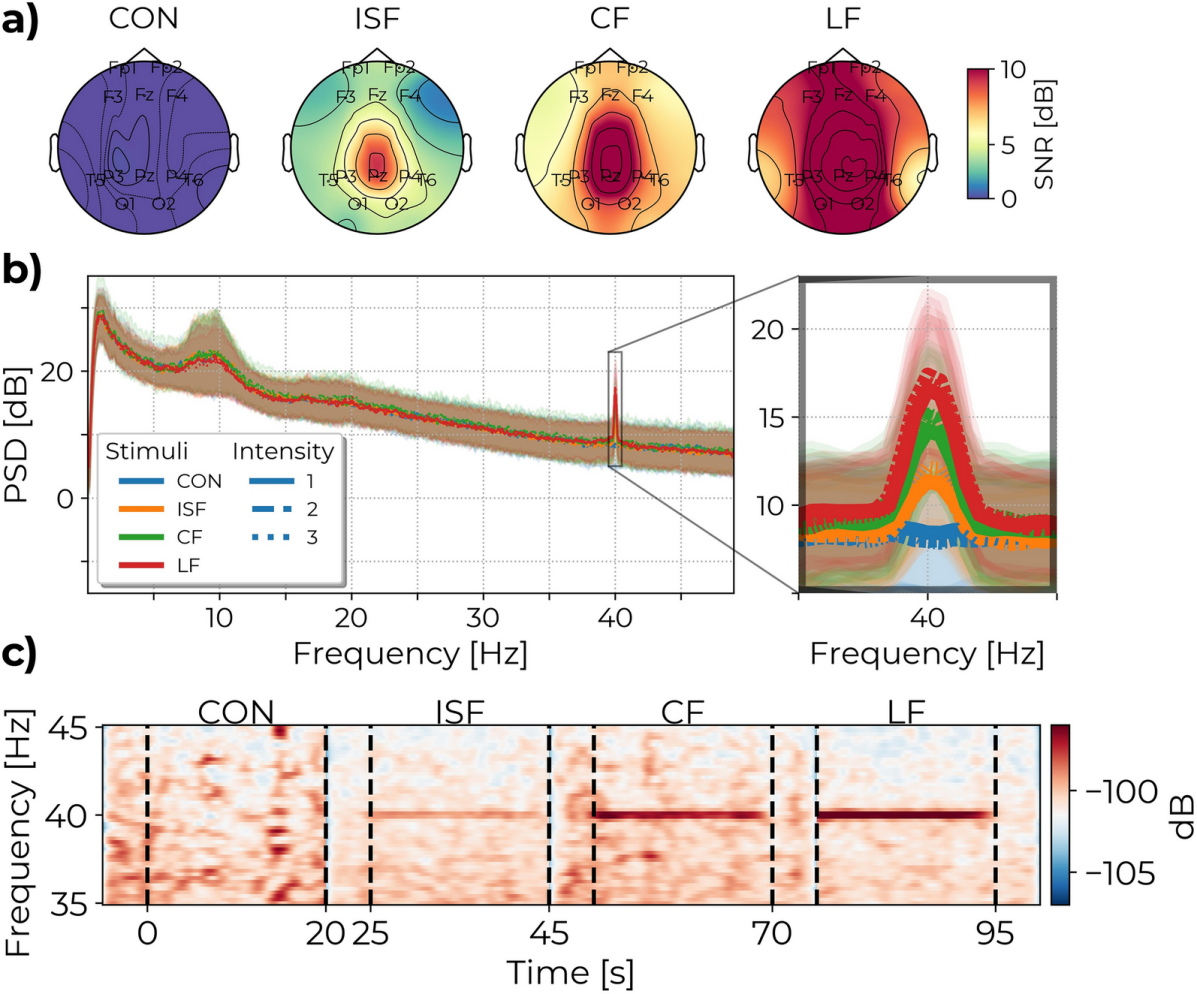
Topographic maps, spectrogram, and average Power Spectral Density plot for the different stimulus types in experiment A. (**a**) The topographic maps show the spatial distributions of the SNR values across channels. (**b**) PSD shows the SSVEP response for each of the 12 stimulus conditions. (**c**) Spectrograms show the presence of the 40 Hz response during stimulation. Stimuli at the highest brightness levels are used for the topographic maps and spectrograms. PSD and spectrograms have been averaged over channels. Shaded areas of the PSD indicate the standard deviation. Figures are included to visualize the results. Interpolation of noisy channels was applied here for illustration purposes but not in the statistical analysis. Topographic maps for all combinations of stimulus and brightness combinations are available in the Supplementary Material Fig. S6 and S7.

The results of experiment A show that all three types of TLM evoke a significantly higher 40 Hz cortical response than the control (see Figs. 4 and 6a). On average, the distribution of the 40 Hz power across electrodes is greatest for LF and lowest for ISF, and CF is between the two, as evident from the topographic map in Fig. 4a. While the highest 40 Hz power was detected by electrodes in the occipital-parietal region, source reconstruction was not attempted, and thus volume conduction limits information from the spatial distribution of the 40 Hz signal. Instead, the average global 40 Hz power is shown for each condition as grand-averaged spectrograms in Fig. 4c and grand-averaged power spectra in Fig. 4b. The estimated global average 40 Hz power was significantly higher than the control for all TLM conditions after correction for multiple comparisons using the Sidak method for control of the family-wise error (FWE) (see Table 2). The mean difference (MD) in SNR between LF and control was 9.74 dB (95% confidence interval (CI): 8.79 to 10.69 dB, $P<.0001$), while the MD between CF and control was 7.32 dB (95% CI: 6.36 to 8.27 dB, $P<.0001$), and the MD between ISF and control was 4.31 dB (95% CI: 3.36 to 5.26 dB, $P<.0001$).

Comparing stimulus intensity, the highest 40 Hz cortical response was achieved by the highest intensity, followed by the middle intensity, and finally the lowest intensity, though the effect was not significant (see Table 2). The MD in SNR between the middle and lowest intensity was 0.3 dB (95% CI: -0.5 to 1.0, P = 0.67), while the MD between the highest and lowest intensity was 0.4 dB (95% CI: -0.4 to 1.2, P = 0.44).

Additionally, there were significant subject- and repetition effects, suggesting both inter-subject variability in the strength of cortical 40 Hz response and a high degree of test-retest variability over time (explained variance: $\hat{\sigma }_{{\text{subject}}}^{2} = 1.75$ and $\hat{\sigma }_{{\text{repetition}}}^{2} = 0.12$). Finally, the three-way interaction effect between subject, stimulus, and intensity was significant ($P<.0001$) and explained most of the variance ($\hat{\sigma }_{{\text{stimulus,brightness,subject}}}^{2} = 3.49$), which indicates a subject preference for certain combinations of TLM type and intensity.

These results indicate that LF, CF, and ISF evoke significant 40 Hz power but at varying magnitude, and that the intensity of the light has less of an impact than the choice TLM.

### Experiment B: angles of exposure

**Fig. 5:**
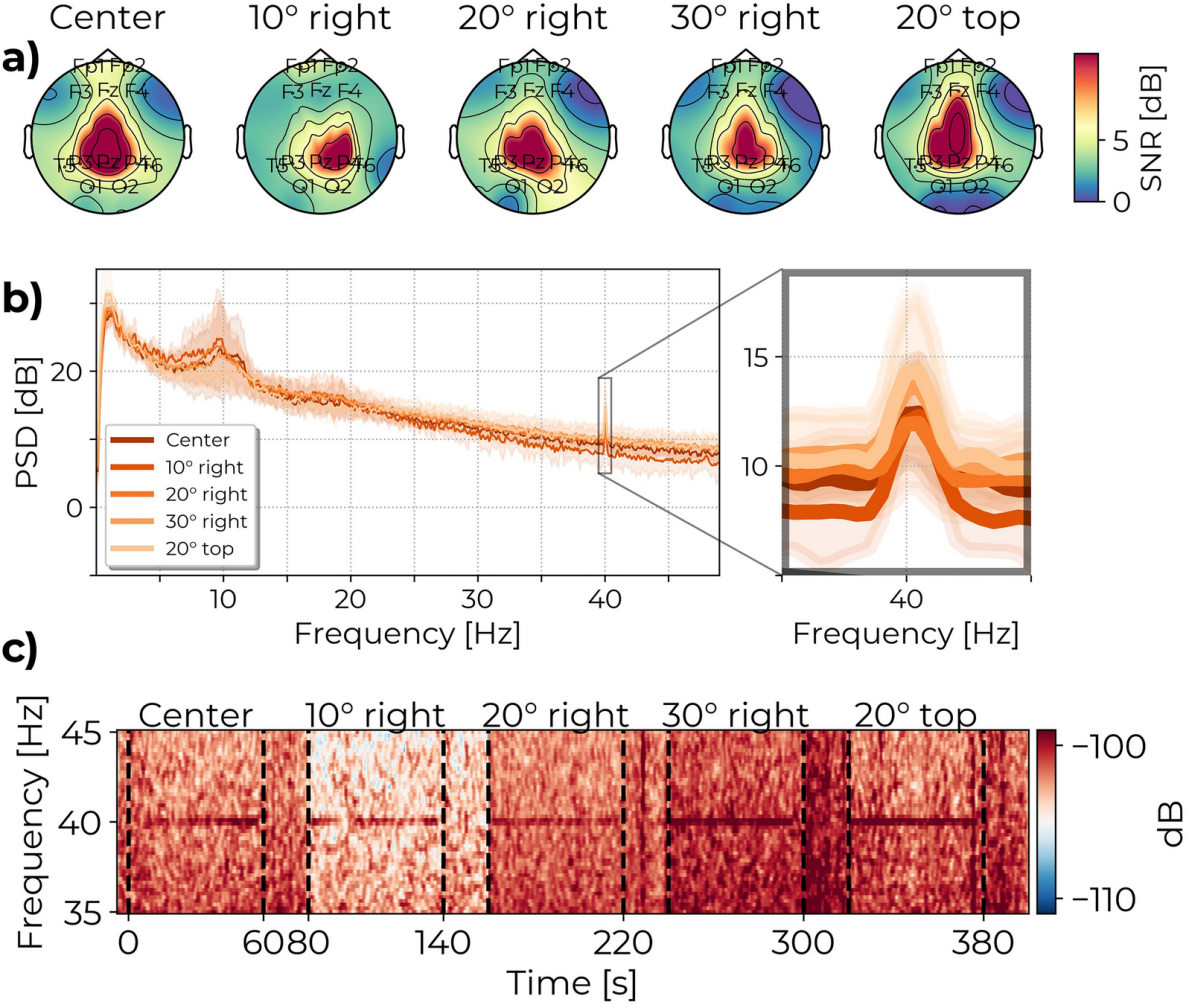
Topographic maps, spectrogram, and average Power Spectral Density (PSD) plot for the different angles of exposure in experiment B. (**a**) The topographic maps show the distribution of the SNR values in each channel. (**b**) PSD shows the SSVEP response for each of the exposure angles. (**c**) Spectrograms show the presence of the 40 Hz response at all angles of exposure. PSD and spectrograms have been averaged over channels. Shaded areas of the PSD indicate the standard deviation. Figures are included to visualize the results. Interpolation of noisy channels was applied here for illustration purposes but not in the statistical analysis.

**Fig. 6:**
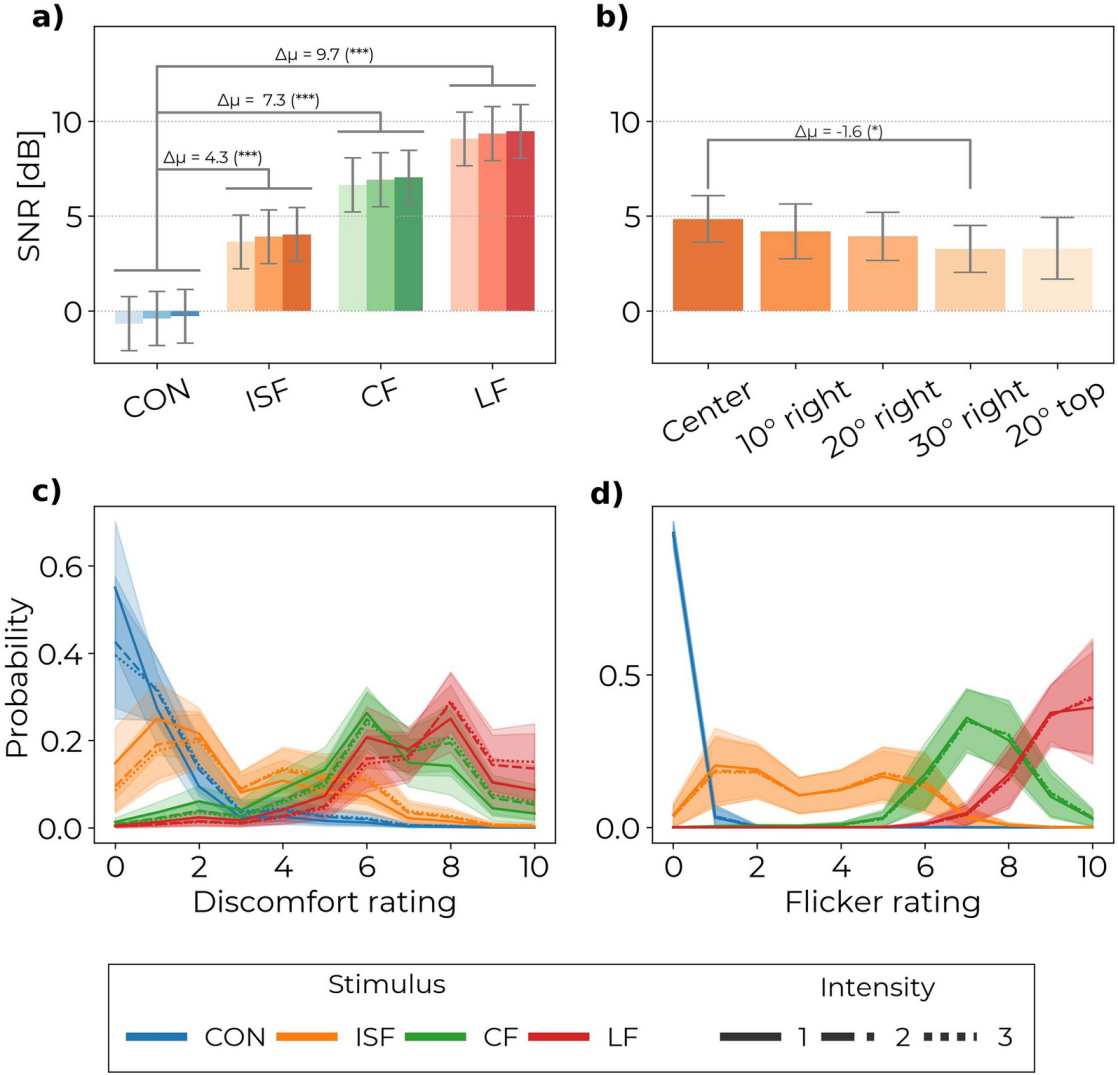
Estimated mean SNR values from experiment A-B and estimated probability of each rating on perceived discomfort and flicker in experiment C. Barplot (**a**) shows the estimated mean SNR values of each stimulus in experiment A and estimated mean difference from CON averaged over the levels of brightness. All light-based gamma stimuli are found to be significantly different from the CON light. The level of brightness was found to be non-significant for the SNR values. Barplot (**b**) shows the estimated mean SNR values of each angle of exposure in experiment B and the estimated mean difference from direct exposure. Exposure from an angle of 30° to the right of the center was significantly different from the direct exposure. A trend of increasing response with increasing brightness and a trend of decreasing response with increasing exposure angle is observed. Mean differences are estimated from the linear mixed effects models after adjustment for multiple comparisons using the Sidak method. Only significant differences are shown in the barplots. The probability of each rating on perceived discomfort (**c**) and flicker (**d**) is estimated from the cumulative linked mixed effects model.

In experiment B, a 40 Hz cortical response is observed for all angles of exposure (see Fig. 5) similarly to the ISF condition in experiment A. The effect of exposure angle on SNR was significant, see Table 1. Only significant contrast was found between the 30 degrees horizontal and central conditions after correction for multiple comparisons (MD: − 1.58 dB, 95% CI: − 2.93 to − 0.23 dB, P = .02). Differences between the other angles and center were: For 10° horizontal (MD: − 0.66 dB, 95% CI: − 2.33 to 1.02 dB, P = .78), 20° horizontal (MD: − 0.91 dB, 95% CI: − 2.32 to 0.494 dB, P = .34), 30° horizontal (MD: − 1.58 dB, 95% CI: − 2.93 to − 0.23 dB, P = .02), 20° vertical (MD: − 1.54 dB, 95% CI: − 3.47 to 0.38 dB, P = .16). The distributions of SNR between stimulation angles presented in Fig. 6b shows the trend for a decrease in 40 Hz SNR with increased exposure angle.

**Table 1:** Post hoc pairwise comparisons for experiment B. Significant mean difference is found for 30° Horizontal exposure compared to the center.

	MD	CI	P	d
10° Horizontal - Center	− 0.66	− 2.3–1.02	0.78	− 0.96
20° Horizontal - Center	− 0.9	− 2.3–0.49	0.34	− 1.33
30° Horizontal - Center	− 1.6	− 2.93 - − 0.23	0.02 (*)	− 2.30
20° Vertical - Center	− 1.5	− 3.45–0.38	0.16	− 2.25

Sidak method is used to estimate the Mean Difference (MD), 95% Confidence Interval (CI), *P*-value (*P*), and Effect size (d).

Significance: * P < .05, ** P < .01, *** P < .001.

The topographic maps in Fig. 5a show similar distributions of 40 Hz power across the electrodes between stimulation angles. While a change in the spatial pattern of cortical activation is possible as a consequence of altering which part of the field of view is stimulated, such nuances are not expected to be evident at the sensor level. Spectrograms in Fig. 5c display only a slight variation in peak 40 Hz power between angles, but also a varying degree of broadband aperiodic noise around the peak. Power spectra in Fig. 5b indicate a similarity of global 40 Hz power between angles above the aperiodic noise. The variation in broadband noise may be an artifact from moving the head position between trials, a matter of test-retest variability and low number of repetitions, and/or between subjects variability from having each subgroup of 10 subjects only be exposed at two of the four non-center angles.

There was a significant subject effect which explained most of the variance ($\hat{\sigma }_{{\text{subject}}}^{2} = 4.33$) suggesting a high degree of inter-subject variability. Additionally, the interaction effect between subject and angle was significant (explained variance: $\hat{\sigma }_{{\text{subject,angle}}}^{2} = 2.17$), indicating that subjects were affected differently by the change in exposure angle, perhaps by an uncontrolled confound of modulated visual attention.

Furthermore, Bayesian ANOVA showed extreme evidence that the cortical 40 Hz power from all angles of exposure was significantly higher than the control stimulation (BF $\gg$ 100, see Supplementary Material Table S4).

The decline in SNR is expected both from the reduced flux of light through the pupil at increased angles and potentially reduced visual attention to the stimulus. The result implies a trade-off between the 40 Hz SNR and the geometric position of the light source, and disengaging from gazing directly at the stimulus comes at a cost of slightly lower evoked 40 Hz cortical power. However, the presence of a cortical 40 Hz response even at high exposure angles indicates that it is a viable alternative to direct stimulation.

### Experiment C: discomfort and flicker

In experiment C, the discomfort rating was significantly increased for all three types of TLM compared to control (see Table 2). Figure 6c shows the probability of each discomfort rating for each of the conditions. Of the three TLMs, ISF had the lowest discomfort score with an increase from control of 1.9 (95% CI: 1.4 to 2.4, $P<.001$), while CF scored 5.1 higher discomfort than control (95% CI: 4.7 to 5.6, $P<.001$), and LF scored 6.2 higher than control (95% CI: 5.8 to 6.6, $P<.001$). These results suggest that all types of TLM are more uncomfortable than static light, though not to the same degree. The CF was significantly more uncomfortable than ISF (MD: 3.2, 95% CI: 2.7 to 3.7, $P<.001$), and so was LF (MD: 4.3, 95% CI: 3.9 to 4.8, $P<.001$). Finally, LF was significantly more uncomfortable than CF (MD: 1.1, 95% CI: 0.7 to 1.6, $P<.001$). As such, the most comfortable choice among the three is ISF whose score was closer to that of the control than both CF and LF. The discomfort rating was also significantly affected by brightness though to a lower degree than the type of TLM (see Table 2). As expected, the lowest discomfort was reported for the lowest brightness and the highest for the highest brightness setting. The middle brightness was significantly more uncomfortable than the lowest (MD: 0.5, 95% CI: 0.2 to 0.8, $P<.01$), and so was the highest brightness with a difference to the lowest brightness of 0.6 (95% CI: 0.2 to 0.8, $P<.001$). The middle and highest brightness were not significantly different (MD: 0.1, 95% CI: − 0.2 to 0.5, P = 0.65).

In terms of perceived flicker, all three types of TLM scored significantly higher than the control (see Table 2). Figure 6d shows the probability of each flicker rating for each of the conditions. Of the three TLMs, ISF scored the lowest on perceived flicker at 3.4 higher than control (95% CI: 2.7 to 4.1, $P<.001$), while CF scored 7.2 higher than control (95% CI: 6.2 to 7.6, $P<.001$), and LF scored 9.1 higher than control (95% CI: 8.8 to 9.4, $P<.001$). These results suggest that all types of TLM are perceived to be more flickering than static light, though at vastly different degrees. Both CF and LF scored significantly higher than ISF in terms of perceived flicker. The flicker score was 3.9 higher for CF than ISF (95% CI: 3.4.8 to 4.4, $P<.001$) and 5.7 higher for LF than ISF (95% CI: 5.2 to 6.2, $P<.001$). The brightness did not significantly impact the degree of perceived flicker (see Table 2 and Fig. 6d).

**Table 2:** Post hoc pairwise comparisons for experiments A and C. Estimate mean difference compared to the control condition. Mean differences, confidence intervals, and P-values are estimated using the Sidak method for correction of multiple comparisons. Results show significant differences in both discomfort and flicker between all the light-based gamma stimuli (ISF, CF, and LF) and CON. Significant differences between the lowest levels of brightness (1) and the remaining levels (2 and 3) are also found for the perceived discomfort but not for the perceived flicker. Significant differences in both discomfort and flicker are also found between ISF and the two other light-based gamma stimuli (CF and LF). No significant interaction effect between stimulus and brightness was found for either perceived discomfort or flicker.

	SNR				Discomfort				Flicker			
	MD	CI	P	d	MD	CI	P	d	MD	CI	P	d
Stimulus												
ISF-CON	4.3	3.4–5.3	(***)	2.1	1.9	1.4–2.4	(***)	2.8	3.4	2.7–4.1	(***)	3.5
CF-CON	7.3	6.7–8.3	(***)	3.6	5.1	4.7–5.6	(***)	7.6	7.2	6.8–7.6	(***)	7.5
LF-CON	9.7	8.8–10.7	(***)	4.8	6.2	5.8–6.6	(***)	9.2	9.1	8.8–9.4	(***)	9.4
CF-ISF	—	—	—	—	3.2	2.7–3.7	(***)	4.8	3.9	3.4–4.4	(***)	4.0
LF-ISF	—	—	—	—	4.3	3.9–4.8	(***)	6.4	5.7	5.2–6.2	(***)	5.9
LF-CF	—	—	—	—	1.1	0.7–1.6	(***)	1.6	1.8	1.6–2.0	(***)	1.9
Brightness												
2–1	0.3	− 0.5–1.0	0.67	0.1	0.5	0.2–0.8	0.001 (**)	0.75	0.07	− 0.15–0.3	0.74	0.07
3–1	0.4	− 0.4–1.2	0.44	0.2	0.6	0.3–1	(***)	0.94	0.09	−0.1–0.3	0.60	0.10
3–2	—	—	—	0.06	0.12	− 0.2–0.5	0.65	0.12	0.02	− 0.2–0.2	0.97	0.02

Sidak method is used to estimate the Mean Difference (MD), 95% Confidence Interval (CI), P-value (P), and Effect size (d).

Significance: *P < .05, **P < .01, ***P < .001. Entries marked with — are not tested within the family of hypotheses.

### Correlation between flicker, discomfort, and entrainment

**Fig. 7:**
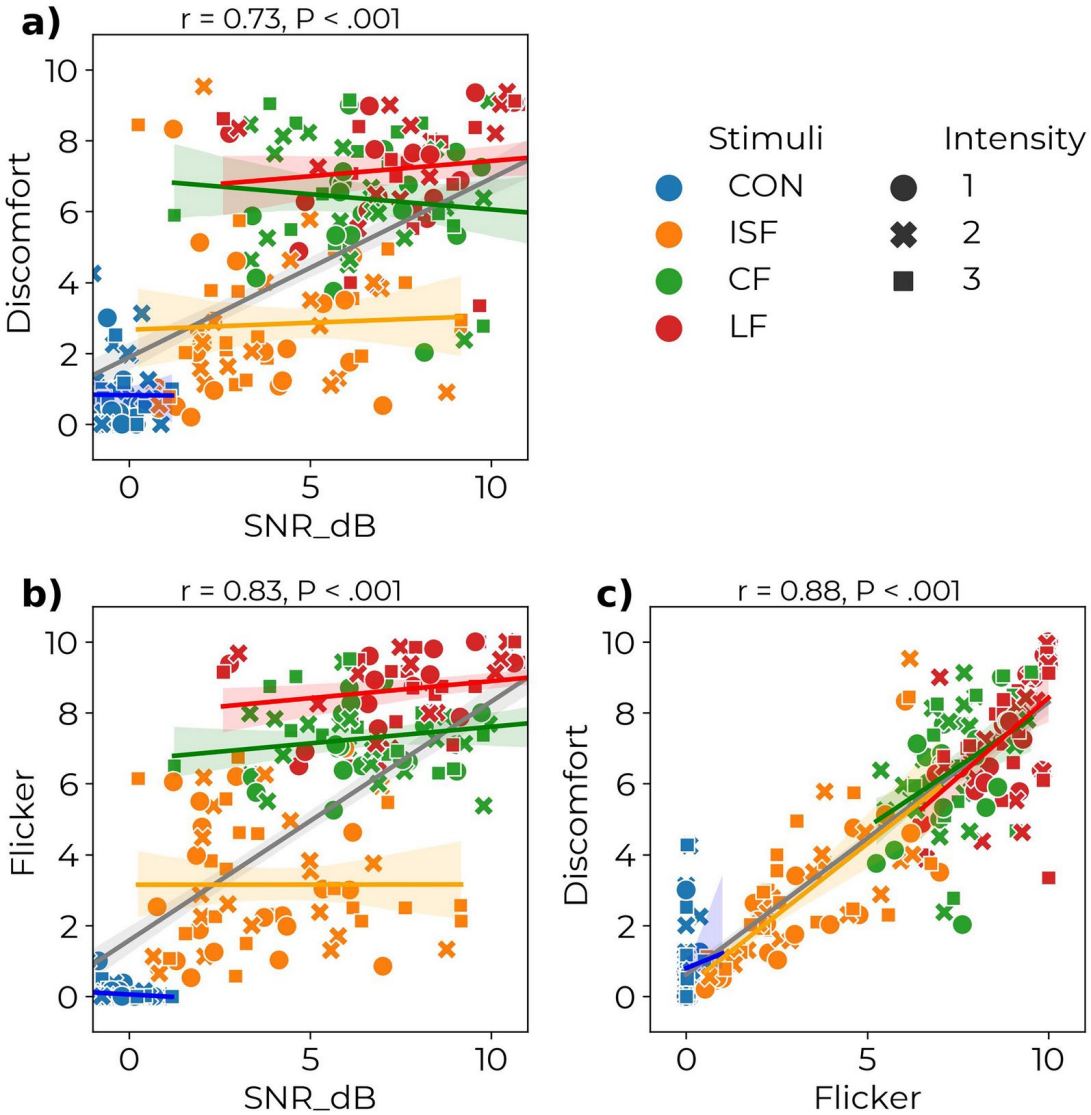
Correlation between neural entrainment and visual perception. Scatterplot and Spearman’s correlation coefficient between (**a**) the 40 Hz SNR and experienced discomfort, (**b**) the 40 Hz SNR and the experienced flicker, and (** c**) the experienced flicker and experienced discomfort for each type of stimulus. r denotes Spearman’s correlation coefficient for the aggregated data, and *P* is the *P*-value of the significance of the correlation. A significant positive correlation is found for all pairwise correlations. The scatterplots show observations from experiments A and C for each subject averaged over repetitions and EEG channels and averaged over repetitions, respectively. Linear regression models are fitted to the entire data and to each stimulus type (ISF, CF, IF) to estimate the best fit, and these lines are included in the scatterplots.

When evaluating the results of experiments A, B, and C, there appears to be a correlation between the SNR for the 40 Hz cortical response to stimulation, the degree of perceived flicker from the stimuli, and the amount of experienced discomfort from the stimuli. To further foster the discussion on the feasibility of gamma stimulation, an exploratory analysis of the correlation structure was conducted using the non-parametric Spearman’s correlation. Figure 7 shows the pairwise correlations between perceived discomfort, flicker, and the 40 Hz SSVEP SNR. A significant ($P<.001$) positive correlation exists between both perceived flicker and perceived discomfort (r = 0.88) and between the 40 Hz SNR and the perceived flicker (r = 0.83) and the perceived discomfort (r = 0.77). This indicates that higher flicker is significantly related to higher discomfort and that a higher perception of flicker and discomfort also is related to higher acute gamma entrainment. In practice, this means that there might be a trade-off between comfort and efficacy and that a balance must be struck to find the optimal visual stimulation implementation.

When the correlations are estimated individually for each type of stimulus, the results are more nuanced. The individual slopes for the correlation between perceived flicker and experienced discomfort are almost identical to the aggregate correlation slope (see Fig. 7c). This is supported by a cumulative mixed model indicating that experienced discomfort is explained only by the perceived flicker. However, the individual slopes for the correlation between SNR and discomfort are different from the aggregated correlation (see Fig. 7a). The cumulative mixed model also indicates a significant main effect of stimulus and an interaction between stimulus and SNR. Analysis of the perceived flicker (Fig. 7b) shows similar results. These findings imply that within a given choice of TLM, an increase in SNR is associated with a slight increase in perceived flicker or experienced discomfort.

## Discussion

In this study, we compared various visual GENUS options for entraining a 40 Hz brain response to assess their acute neural response and feasibility. This was done by measuring the acute 40 Hz brain entrainment and visual perception scores (perceived flicker and visual discomfort). Additionally, for further exploration of real-world application, we measured the acute 40 Hz brain entrainment under peripheral exposure by facilitating an experiment with different gazes increasing further from the center of the stimulation device.

The results showed a clear correlation between the 40 Hz entrainment and the subjective perception of discomfort and flicker. Higher entrainment is followed by higher discomfort and perception of flicker (see Fig. 7). Luminance and chromatic flicker result in the highest 40 Hz entrainment power but are also perceived as the most flickering and experienced as the least comfortable. In contrast to this, ISF stimulation results in the lowest entrainment power, though significant compared to control stimulation, and with significantly better ratings in perceived flicker and comfort than LF and CF. We propose that ISF stimulation is a more feasible option for 40 Hz visual stimulation as a potential treatment because of its tolerability during 40 Hz entrainment. This is supported by the comparatively lower drop-out frequency and higher treatment adherence in clinical trials using ISF^99^ than combined audiovisual stimulation^53^.

We find that lowering stimulation brightness significantly lowers the discomfort but not the perceived level of flicker. Stimulation at brightness levels, from 5,200 to 10,400 lux, entrain a significant 40 Hz response, though a trend towards decreased entrainment is observed with lower brightness. This trend agrees with previous findings^49,70^. Given that discomfort glare is affected by intensity but also highly subject-dependent^100,101^, these results suggest that users may benefit from an option to individually select the stimulation intensity for improved tolerability. It is important to note the effect sizes are relatively small and therefore higher sample size is required to confirm these results.

Increasing the stimulation angle emulates a treatment setting in which the user can partake in other activities while receiving stimulation passively. Despite a decrease in response with increased stimulation offset, the difference is non-significant up to 20 degrees horizontal and vertical offsets, compared to no offset. The immediate benefit of this is the prospect of users introducing the treatment into their existing habits with minimal effort. An additional analysis comparing the estimated mean values of the control stimulation in experiment A and the estimated mean values for each angle of exposure in experiment B shows evidence that there is a significant response at all angles.

Together, these findings present several trade-offs. The first is a trade-off between comfort and entrainment power. The second is a trade-off between perceived flicker and entrainment power. The third is a trade-off between brightness and entrainment power. Finally, we show a trade-off between stimulation angle and entrainment power. These trade-offs give rise to optimization problems whose Pareto fronts will be valuable to explore.

It is not yet clear how the acute entrainment power affects the medical response in AD, or if other markers are more relevant for the neuroprotective effects presented in^39,46–48^. Such markers could include the duration of entrainment above a certain power threshold, the spatial distribution of the entrainment, or a complex combination of either. Under the conservative assumption that the medical benefit is highly correlated with the entrainment power, it may still be favorable to choose a more comfortable treatment option if it leads to better adherence. Adherence pertains both to the selection of stimulus type and stimulation intensity, duration, and exposure angle parameters. By adjusting factors like brightness and angle of exposure, and even frequency or color settings, while measuring the neural entrainment it will be possible to design personalized treatment options for each individual patient. Results indicate general trends that highlight the complexity and high inter-subject variability of visual brain stimulation. Optimization schemes for the AD population should be designed not only by the acute neural response alone but also based on user feedback, attention scores, and disease progression. A possible approach could be to employ Bayesian optimization and neurofeedback techniques.

Across several medical subfields, including AD, increased treatment side effects are related to lower adherence^102,103^. While several light-based GENUS paradigms are suggested for AD treatment, no study has (to the author’s knowledge at the time of submission) compared them in terms of tolerability. Our results indicate that the potential treatment barriers to GENUS for AD may be overcome by carefully engineered light technologies designed for higher comfort and feasibility. Considering the traits of patients with AD, a 1-hour daily treatment session might be infeasible due to the impaired attention associated with this population^104^. Our findings contribute to the design and use of new treatment options by providing evidence of the effect of different stimulation, brightness, and angles of exposure. Results suggest that visual brain stimulation treatment can be administered passively, potentially during other daily activities, and that alternative methods than luminance flicker exist. It is important to note that these results should be confirmed in longitudinal studies ideally by tracking adherence followed by a stratified analysis of different groups. Agger et al.^51^ propose gaze-tracking as a measure of adherence and classification of direct and indirect exposure. Other approaches could include complementary EEG recordings that validate the neural entrainment during stimulation.

We recognize that this study’s participants were limited to healthy young volunteers and may not accurately reflect the AD population for which this type of treatment is intended. Findings from this experiment, however, are in agreement with those of Agger et al.^91^. Their main EEG findings showed a significant response from both ISF and LF (STROBE) compared to CON with the highest response achieved by LF. However, further research is warranted within this field. Both the elderly and AD populations may differ from the healthy young population in terms of retinal sensitivity to light, attention levels, neural adaptations, and synchronization with the stimulation frequency. These are all critical factors to consider when evaluating the feasibility of new treatments. Individual variance even occurs in young and healthy populations in terms of sensitivity to light, attention, and general cognitive readiness. Our findings support this as we see a significant effect and variability between the participants. Individuals with light sensitivity may exhibit lower SSVEP responses due to discomfort-induced physiological behaviors, such as squinting or blinking, potentially decreasing signal amplitudes. However, their responses might also be higher overall, influenced by a lower threshold for visual stimulation. Similarly, visual stimulation combined with a simultaneous visual attention task or cognitive load^68,105^ has proven to positively modulate the SSVEP response in the gamma range, both in terms of power and spatial propagation. Such findings should be considered when evaluating the effectiveness of stimulation, as some types of stimulation can more easily be combined with additional tasks than others. Confunding factors of attention and cognitive load would be interesting to investigate in further research by designing experiments and analyses that include attention or cognitive paradigms.

Future research should expand the study to include a more diverse population, including elderly participants and those with AD, to better understand the generalizability of the findings and assess the potential impact of individual differences in light sensitivity, attention levels, and cognitive function.

This study provides further evidence to support the potential of gamma entrainment therapies for AD treatment. We have shown that ISF stimulation can effectively entrain 40 Hz brain activity while being significantly more comfortable and tolerable than other methods. Additionally, we have demonstrated the feasibility of achieving entrainment from passive exposure, suggesting that ISF stimulation could be a convenient and practical treatment option for AD.

## Supplementary Information

Supplementary Information.

## Data Availability

Data can be shared upon request for scientific purposes that do not violate the data processing agreement. Contact the authors for more information.
