# Supplementary material for: Light-based gamma entrainment with novel invisible spectral flicker stimuli
Source: Sci Rep. 2024 Nov 29;14:29747. doi: 10.1038/s41598-024-75448-4 (PMC11606973; doi:10.1038/s41598-024-75448-4)
Supplement: Supplementary file 1 — Supplementary Information. [file 41598_2024_75448_MOESM1_ESM.pdf]

Supplementary Information

Study Name: ALZLIGHT EEG Study: Study of How Spectral Quality and Brightness of Flickering and Non-Flickering Visible Light Modulates EEG Responses

Study Protocol: Approved by the Institutional Review Board at DTU Compute, Technical University of Denmark (*application number: COMP-IRB-2020-01, approved 14-02-2020*).

Manuscript title: Light-Based Gamma Entrainment with Novel Invisible Spectral Flicker Stimuli

Authors: Luna S. Hansen (1), Marcus S. Carstensen (2), Mark A. Henney (3), N. Mai Nguyen (4), Martin W. Thorning-Schmidt (5), Jes Broeng (6), Paul Michael Petersen (7), and Tobias S. Andersen (8)

Contact information:

1) lunash94@hotmail.com, +45 20 44 60 29
2) mascca@dtu.dk, +45 23 45 64 42
3) maalhe@dtu.dk , +45 29 92 89 95
4) mai@optoceutics.com,
5) martinwts@gmail.com
6) jesbo@dtu.dk, +45 24 25 38 35
7) pape@dtu.dk +45 46 77 45 12
8) toban@dtu.dk +45 45 25 36 87

Contents

Supplementary Method: 3
Recruitment 4
Data Acquisition 5
EEG system 5
Light assessment 6
Light settings 7
Luminance Matching 8
Data 9
Data processing 10
Statistical design and analysis 11
Supplementary Equations 12
Full (initial) model for experiment A: 12
Full (initial) model for experiment B: 12
Full (initial) model for experiment C: 12
Supplementary Tables 13
Post hoc analysis – Experiment A 13
Post hoc analysis – Experiment B 14
Post-hoc analysis: Discomfort 15
Post-hoc analysis: Flicker 15
Probability statistics – Experiment C 16
Supplementary Figures 17
SNR Spatial distribution of brightness levels 18

List of Supplementary Figures

Figure S1 | Rating scale presented to the participants during experiment C. 6
Figure S2 | Illustration of the luminance matching between the 4 different light stimulus at the highest brightness level in exp. A. 8
Figure S3 | Light settings. Top: Spectral composition of the 2 sets used for ISF (right) and CF (left) along with the spectrum of combining the two sets. The combined spectral corresponds to the spectral of STROBE and CON. Bottom: the 1931 CIE diagram and the coordinates of all the light stimulus. 8
Figure S4 | Participant recruitment flowchart. 9
Figure S5 | Data processing pipeline 10
Figure S6 |Brightness Spatial Response on Common Axis: Row 1: CON, row 2: ISF, row 3: CF, row 4: LF. 17
Figure S7 | Brightness Spatial Response Individual Axis: Row 1: CON, row 2: ISF, row 3: CF, row 4: LF. 18

List of Supplementary Tables

Table S1 | Post-hoc comparisons of the SNR values from the different light stimulus in experiment A. Note STROBE refers to Luminance Flicker (LF) 13
Table S2 | Post-hoc comparisons of the SNR values from the different levels of brightness in experiment A 13

Table S3 |Post-hoc comparisons of the SNR values from the different exposure angles in experiment B 14
Table S4 | Bayesian ANOVA - Post Hoc Comparisons of data from experiment B and the control setting in exp. A. 14
Table S5 | Post-hoc comparisons of the discomfort ratings from the different light conditions in experiment C. Note STROBE refers to Luminance Flicker (LF).. 15
Table S6 | Post-hoc comparisons of the flicker ratings from the different light conditions in experiment C. Note STROBE refers to Luminance Flicker (LF).. 15
Table S7 | The 12 light-based stimuli and rating with the highest probability estimated by cumulative linked mixed effects model. Note STROBE refers to Luminance Flicker (LF).. 16

Supplementary Method:

The following text is taken from the study protocol approved by Institutional Review Board at DTU Compute.

" … the study aims at investigating the SSVEP response and discomfort rating in relation to the type of light and the intensity of the light. Two main dependent measures of interest in the stage II study; the EEG SSVEP response and the discomfort rating during the different light stimuli. The experiment therefore includes two parts. The independent variable of both parts is the type of light stimulation and the intensity. …

The first part [referred to as experiment A in manuscript] will record the SSVEP response during stimulation with 12 different light stimuli: 4 types at 3 levels of intensity each. These will be presented in a randomized order. The recording initially consists of a baseline recording of one minute, following 6 repetitions of 12 runs each of 20 seconds stimulus and 5 seconds' rest. The baseline recording is performed under eyes open condition and no stimulus. The participants are instructed that 12 lights will be presented. However, to prevent any bias, the participant cannot be aware of the detailed configuration of the lights. Gaze tracking can also take place during the EEG recording in order to evaluate compliance.

After this EEG recording 10 additional minutes of EEG will be recorded [referred to as experiment B in manuscript] with eight times 60 seconds stimuli and 20 seconds rest between. The stimuli include the participant looking at a ISF LED configuration from four different angels with 2 repetitions.

In the second part [referred to as experiment C in manuscript] the participant is asked to rate the level of discomfort of each of the 12 different light stimuli. A paradigm inspired by the VAS scale is designed using Python that presents the participant with a stimulus and an interface that prompts the participant to rate according to the following statement: "*How much discomfort did you experience from the stimulus?*", "*How much flicker did you experiences*" and "*How did you like the color*". The participant will rate on a scale from 0-10. 0 being "Minimum discomfort/flicker/pleasant", 5 being "Moderate discomfort/flicker/pleasant" and 10 being "Maximum discomfort/flicker/pleasant". After rating the participant can continue the session and proceed to the next stimulus. Each stimulus will be presented 4 times in a randomized order.

The total length will be around 32 + 8 minutes for the EEG part and between 15-20 minutes for the assessment according to the individual time spent on rating. The second part is conducted after the first part to make the following ranting easier for participant. The order of the two parts is thought to provide the participant with a sense of "what is the worst light" before rating. "

Recruitment

Participants were recruited through the authors' network at the Technical University of Denmark (DTU) (Anker Engelunds Vej 1, Bygning 101A, 2800 Kongens Lyngby, Denmark). Participants were selected based on exclusion criteria, excluding those above the age of 50 and individuals with a history of light sensitivity, neurological or psychiatric diseases, epilepsy, or familial history of epilepsy. Participants underwent a self-reported assessment guided by questions from the investigator. Questions included:

- Do you or anyone in your close family suffer from epilepsy?
- Do you have any neurological or psychiatric disorders?
- Have you ever had any discomfort caused by light?

Recruitment was carried out by a non-medical researcher. And participants were excluded if any doubt on the above was raised or suspected.

Data Acquisition

EEG system

All experiments were conducted in a dedicated EEG laboratory designed for optimal data collection. To minimize electrical interference, all non-essential electronic devices were disconnected, and ambient lighting was reduced. The sole powered device, the VSS 1.0 stimulus system, was connected using a shielded cable. Participants were seated in a comfortable chair approximately 60 cm from the stimulus device, with semi-blinded windows. Strict instructions were given to participants to remain still and relaxed during EEG recordings.

EEG data were acquired using a Zeto Inc. FDA-approved wireless headset with dry electrodes. The headset followed the international 10-20 system electrode placement, with the inclusion of the following channels for subsequent data analysis: Fp1, Fp2, Fz, F3, F4, T5, T6, P3, P4, Pz, O1, and O2. Ground was established at Fpz, and linked mastoids served as the reference. Data were sampled at a rate of 500 Hz using dedicated software. The EEG headset included 19 electrodes, but only the frontal, parietal and occipital electrodes were proper recorded and used in the analysis.

The Zeto Inc. EEG headset specifications are as follows:

- Number of electrodes: 19
- Reference electrodes: A1/A2 or built-in choices
- Channel Positions: 10-20 system compliant
- Weight: < 650g or 23oz
- Battery: Rechargeable Li-ion
- Run time: 5-6 hours continuous
- Bandwidth: 0.003 – 250Hz
- Sampling Rate: 500Hz
- Analog to Digital Converter: 24 bits
- Common Mode Rejection Ratio: > 120 dB
- Input Impedance: 1 TΩ
- Noise (1-50Hz): < 1μV RMS
- Wireless Mode: WiFi
- Range: 10m (direct line of sight)

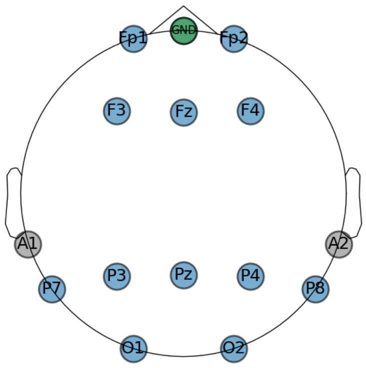

Light assessment

The administration and recording process of the Visual Analogue Scale (VAS) assessments require further elucidation. Participants were instructed to evaluate each stimulation based on the three specified assessments using an 11-point scale ranging from zero to ten. The assessments were conducted utilizing a PC, with the paradigm programmed in Python. Participants had control over the pace of the assessment, initiating each evaluation by clicking. The resulting data were stored in a CSV format. It is noteworthy that the light remained illuminated during the assessment and underwent changes upon clicking the 'next' button. **Figure S1** shows the rating scaled prompt shown to the participants.

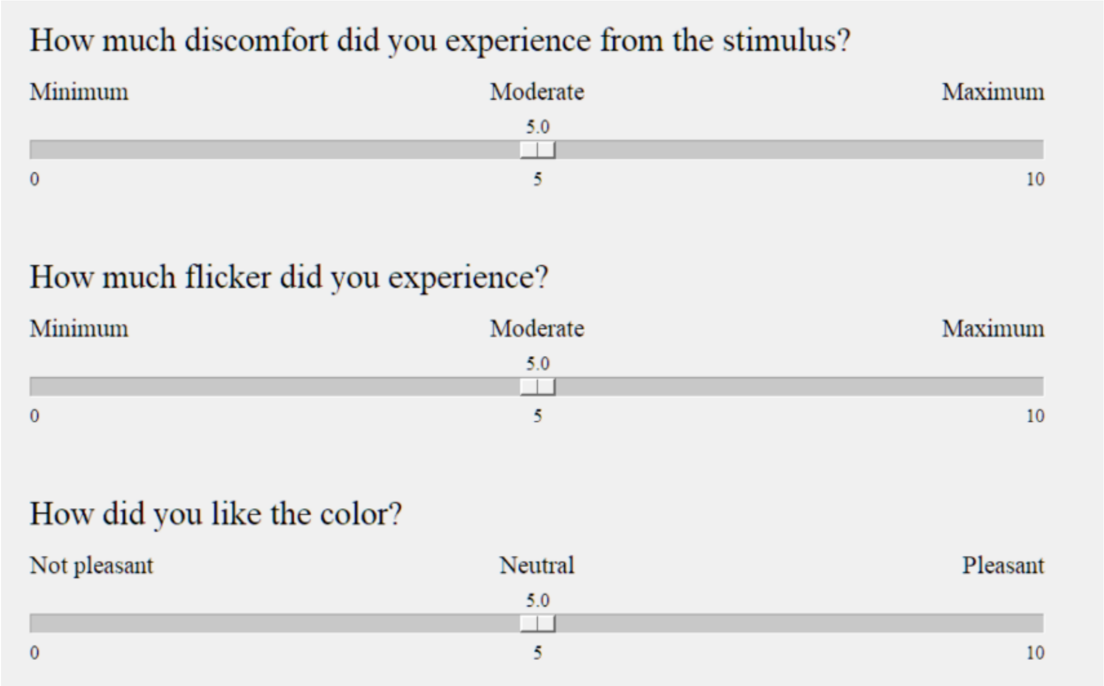

Figure S1 | Rating scale presented to the participants during experiment C.

Light settings

The device is equipped with Multispectral LED array containing six LEDs (blue, cyan, green, lime, amber, red) that can be customized for various light settings. The LEDs are comprised of a mix of direct and phosphor-converted emitters. A specialized LED controller is employed to facilitate precise adjustment of the brightness for each of the six independent channels. This controller also enables forward current control and synchronous timing control for activating/deactivating the different channels. This ensures precise calibration of the 40 Hz stimulation. A modified version of the VSS device using only the blue, red, green, lime, and red LEDs was used for the experiment. Each LED channel is independently controlled, to produce the different types of light output needed.

Dominant wavelengths of the direct emitters and spectral width

- Blue 452.5 nm, HPBW 20.2 nm
- Cyan 499.9 nm, HPBW 34.2 nm
- Green 522.5 nm, HPBW 37.5 nm
- Red 635.7 nm, HPBW 18.0 nm

Dominant wavelengths of the Phosphor converted emitters and their spectral width

- Lime (PC) 567.3 nm, HPBW 108.7 nm
- Amber (PC) 589.7 nm, HPBW 85.2 nm

Device: VSS1.0, LOT0001, SN0037

The figure below is borrowed with permission from *Carstensen, M. S., Lindén, J., Nguyen, N. M., Hansen, H. E., Carrillo, G. M. F., Hansen, L. S., ... & Petersen, P. M. (2020, March). 40 Hz invisible spectral flicker and its potential use in Alzheimer's light therapy treatment. In Mechanisms of Photobiomodulation Therapy XV (Vol. 11221, pp. 47-58). SPIE.*

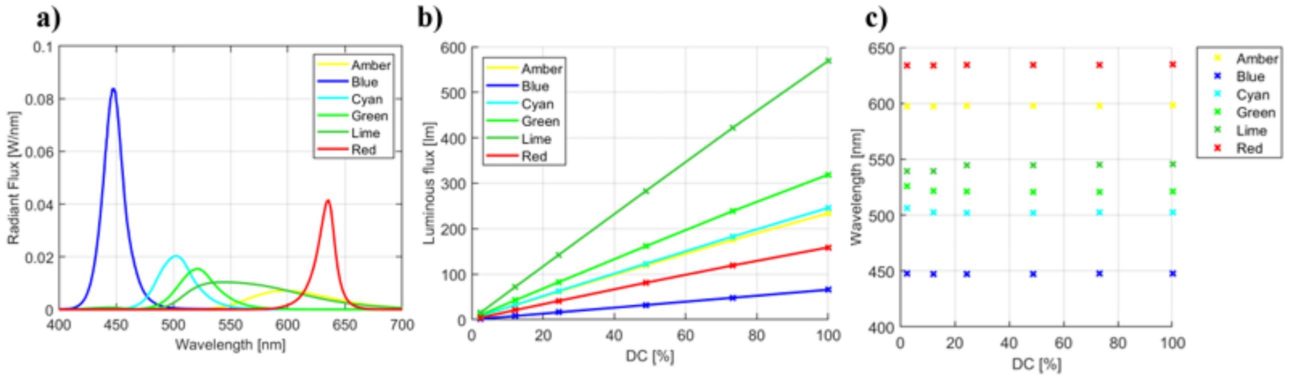

Figure 3. LED Technology Verification. a) Power spectral density plots of all six single-color LEDs. **b)** Linearity of luminous flux output versus duty cycle. **c)** Wavelength stability plot versus duty cycle.

Luminance Matching

Luminance output from the three light settings at the highest brightness level. The luminance using 1931 Color Matching Functions has been measured using a BTS256-EF light-color-spectral meter[1].

To ensure reproducibility the Color Fusion, CF, was created using equal power output in the green and red channel. This increases the perception of flicker, but as flicker is subjective, we thought it would be more consistent to use equal power, instead of subjectively matching. The luminance scale depends on which color matching functions you use, and since they are all subjective scales anyway, we decided on the use of power.

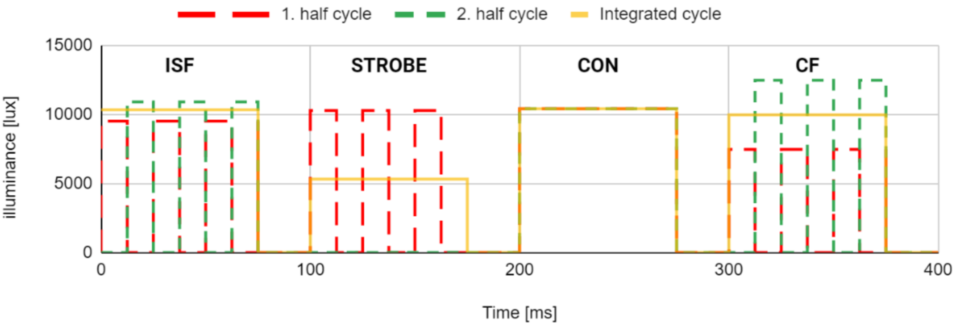

Figure S2 *| Illustration of the luminance matching between the 4 different light stimulus at the highest brightness level in exp. A.*

The spectrum (left) of the two half cycles used for ISF and the spectrum of the combined integrated full cycle used for STROBE (Luminance Flicker, LF) and CON. And the spectrum (right) of the red/green color fused flickering light. Spectrum measured at the surface of the light device using the BTS meter[1]. Position of the different light sets in the CIE diagram.

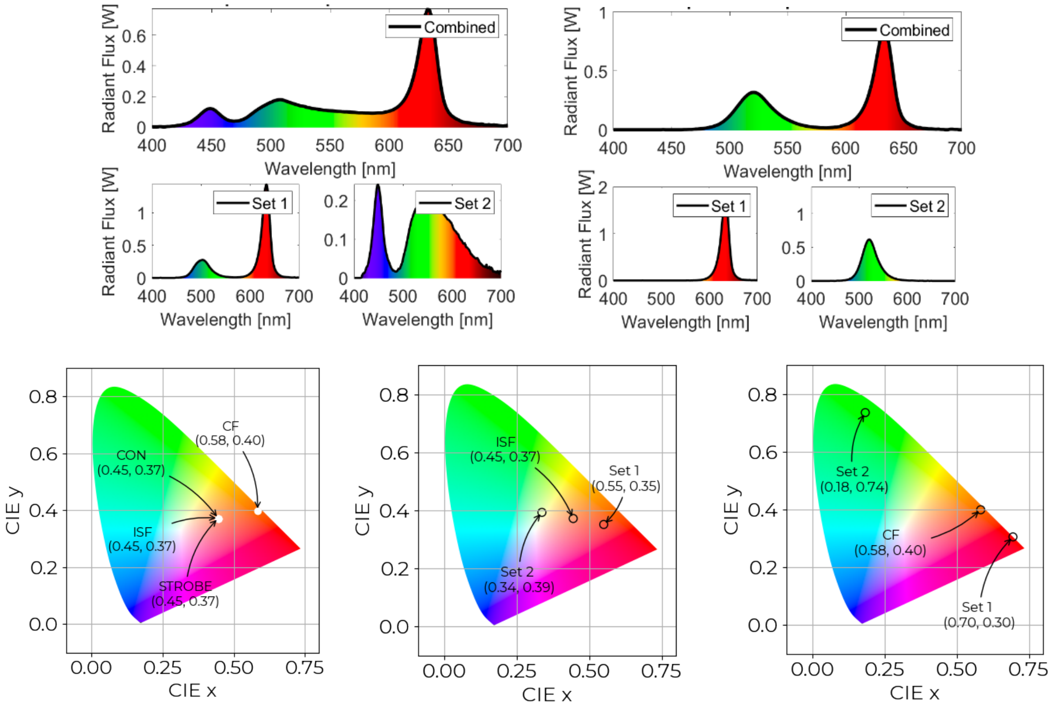

Figure S3 *| Light settings. Top: Spectral composition of the 2 sets used for ISF (right) and CF (left) along with the spectrum of combining the two sets. The combined spectral corresponds to the spectral of STROBE and CON. Bottom: the 1931 CIE diagram and the coordinates of all the light stimulus. Note STROBE refers to Luminance Flicker (LF)*

[1] https://www.gigahertz-optik.com/en-us/product/bts256-ef/

Data

A total of 26 participants were enrolled in the experiment. A single participant was excluded before data collection as he/she did not meet the criteria. 25 participants took part in the experiments. However, due to recording issues only a subset the data could be used. Furthermore, some of the EEG data were excluded due to poor data quality. See **Figure S4** on further details.

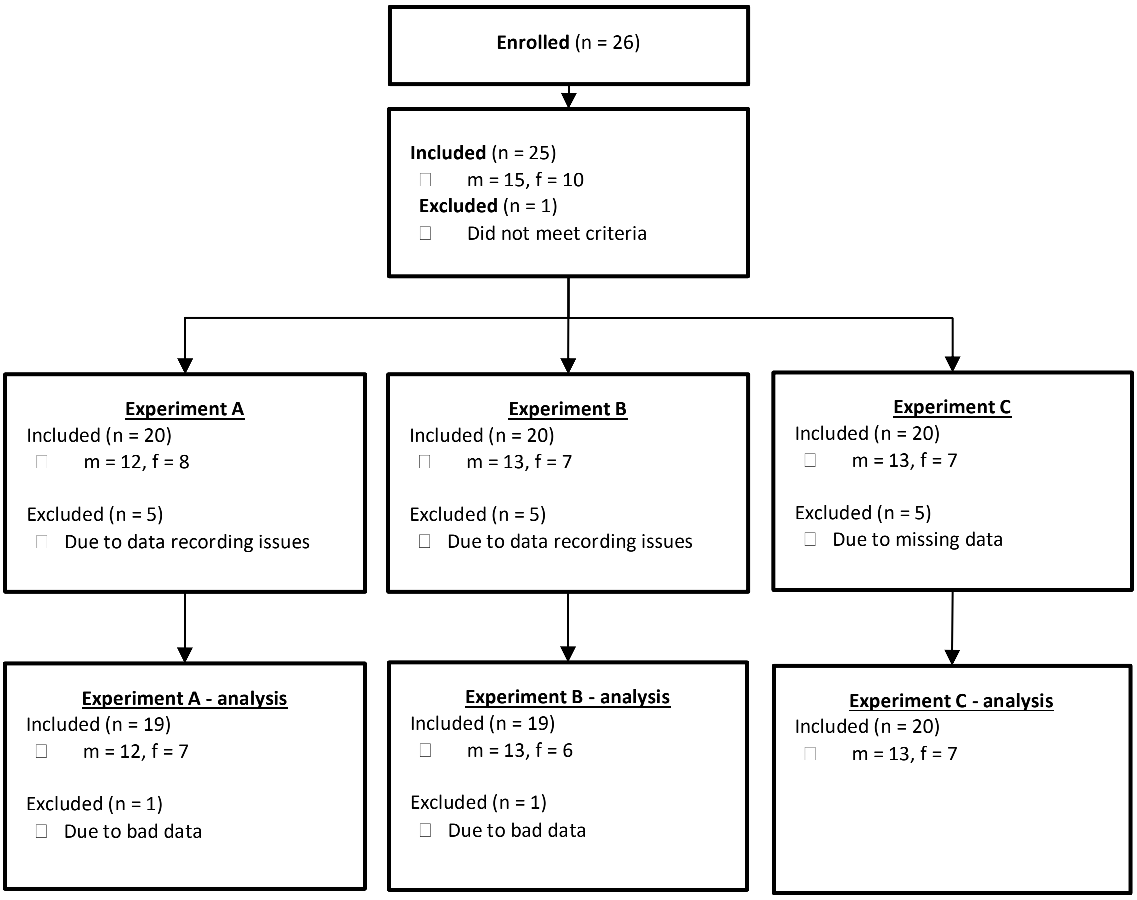

***Figure S4* |** *Participant recruitment flowchart.*

Data processing

Python 3.8 was used to preprocess the EEG data and extract the 40 Hz SNR values. SNR values were imported to R Studio (R4.0) or JASP (0.16.4.0) to perform the statistical analysis.

The library MNE version 0.24.1 from python was used to preprocess the data. The preprocessing and data processing flow can be seen in **Figure S5**. Build-in functions from MNE were used for filtering, re-referencing, ICA, and PSD estimations. SNR estimations were calculated based on this MNE tutorial. Figures are generated from the outputs and plotted using matplotlib and seaborn.

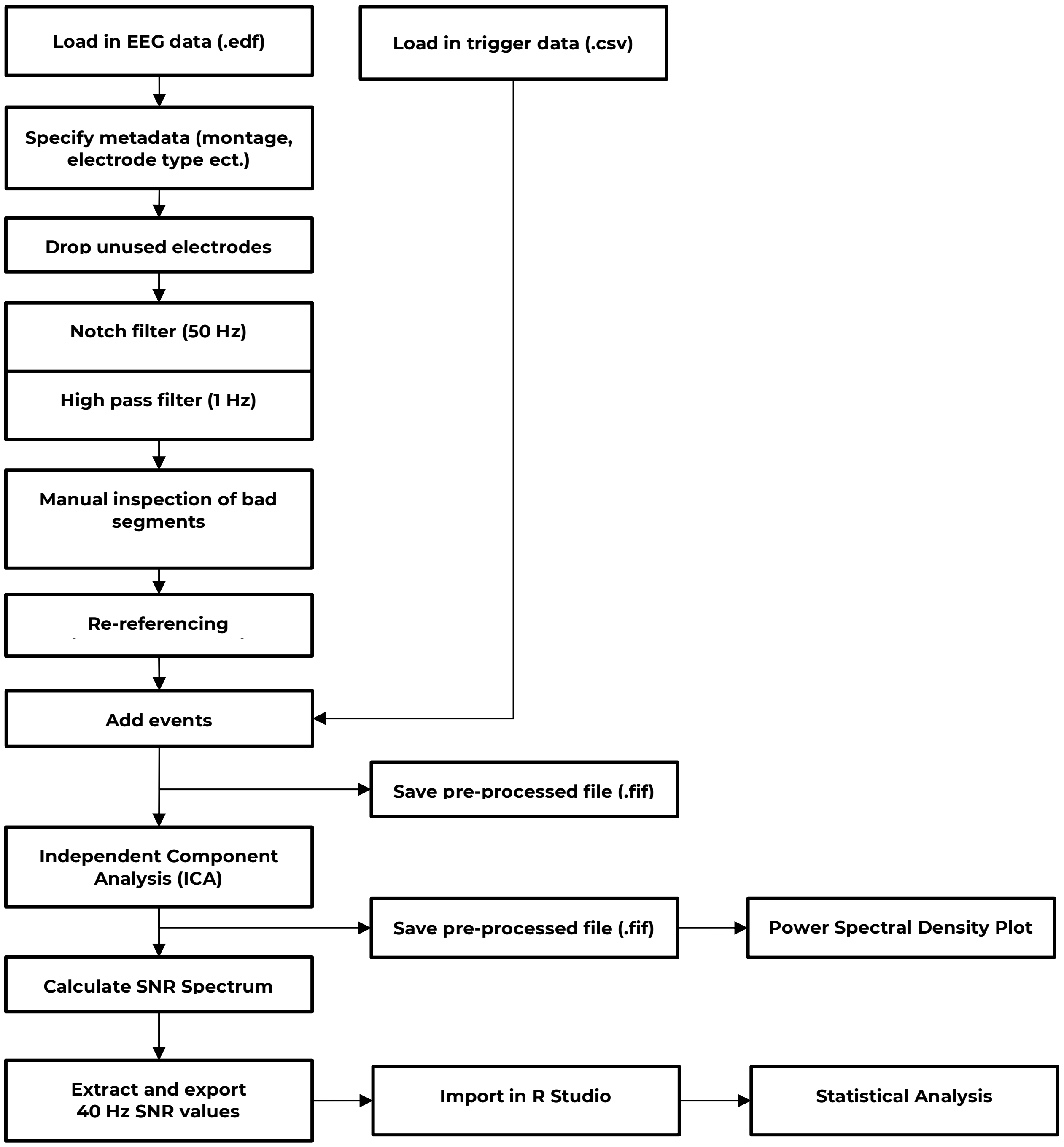

Figure S5 | Data processing pipeline

Statistical design and analysis

For the statistical analysis of the EEG data a linear mixed effects model was applied. For the rating scale data, a cumulative mixed effect model was applied.

The statistical analysis is performed on R using R studio. The analyses were carried on the following steps.

1) Loading data and categorizing data frame values
2) Summary statistics and checking for bias in missing data
3) Exploratory analysis of
    a) Main effect
    b) Interaction effects
    c) Demographic trends
4) Modeling
    a) Checking model assumption
5) Model reduction
6) Post-hoc analysis with correction for multiple comparisons
7) Conclusion

The exploratory analysis aided in determining what parameters to include in the models. All main effects were included. Only interaction effects with indications of a related trend are included. Subjects were included as a random effect in all models. None of the demographic parameters indicated any effect in the exploratory analysis and were therefore not included in the models. The low sample size of each group was also non-representative for any valid conclusion.

Four statistical reports have been prepared and the results of the post hoc analyses can be found in the next sections.

Supplementary Equations

Full (initial) model for experiment A:

Equation (S1):

$$Y_i = \mu + \alpha(stimulus_i) + \beta(brightness_i) + \zeta(stimulus_i, brightness_i) + a(repetition_i) + b(subject_i) + c(repetition_i, subject_i) + c(subject_i, stimulus_i, brightness_i) + e(repetition_i, stimulus_i, brightness_i) + \epsilon_i,$$

where Y_i is the 40 Hz SNR for the *i*th trial and all random variables *x* follows $N(0, \sigma_x^2)$, and $\epsilon \sim N(0, \sigma^2)$

Full (initial) model for experiment B:

Equation (S2):

$$Y_i = \mu + \alpha(angle_i) + a(subject_i) + b(block_i) + c(block_i, subject_i) + d(subject_i, angle_i) + e(block_i, angle_i) + \epsilon_i,$$

where Y_i is the 40 Hz SNR for the *i*th trial, $c \sim N(0, \sigma_{repetition}^2)$, $d \sim N(0, \sigma_{subject}^2)$, and $\epsilon \sim N(0, \sigma^2)$

Full (initial) model for experiment C:

The complete models for experiment C are described by equation (z) in the supplementary equation where the dependent variable, Y, is either experienced discomfort or flicker.

Equation (S3):

$$Y_i = \mu + \alpha(stimulus_i) + \beta(brightness_i) + a(subject_i) + \zeta(stimulus_i, brightness_i) + \epsilon_i,$$

where Y_i is the dependent variable for the *i*th trial, $a \sim N(0, \sigma_{subject}^2)$, and $\epsilon \sim N(0, \sigma^2)$

Supplementary Tables

Post hoc analysis – Experiment A

Stimulus	emmean	SE	df	lower.CL	upper.CL
CON	-0.4523782	0.4361527	43.88293	-1.331452	0.426696
CF	6.8637841	0.4364663	44.01817	5.984154	7.743414
ISF	3.8604025	0.4352161	43.51594	2.983006	4.737799
STROBE	9.2884451	0.4361103	43.86489	8.409446	10.167444

Code

contrast	estimate	SE	df	lower.CL	upper.CL	t.ratio	p.value
CF - CON	7.316162	0.3953216	204.0099	6.364426	8.267899	18.50686	0
ISF - CON	4.312781	0.3939033	201.0255	3.364342	5.261220	10.94883	0
STROBE - CON	9.740823	0.3948732	202.4806	8.790107	10.691540	24.66823	0

Table S1 *| Post-hoc comparisons of the SNR values from the different light stimulus in experiment A. Note STROBE refers to Luminance Flicker (LF)*

Intensity	emmean	SE	df	lower.CL	upper.CL
1	4.667119	0.4133802	35.82230	3.828601	5.505638
2	4.941141	0.4132218	35.76602	4.102899	5.779384
3	5.061929	0.4125521	35.54218	4.224860	5.898998

Code

contrast	estimate	SE	df	lower.CL	upper.CL	t.ratio	p.value
Intensity2 - Intensity1	0.2740221	0.342244	203.5505	-0.4970811	1.045125	0.800663	0.6685238
Intensity3 - Intensity1	0.3948098	0.341531	201.9234	-0.3747330	1.164353	1.156000	0.4360698

Table S2 *| Post-hoc comparisons of the SNR values from the different levels of brightness in experiment A*

Post hoc analysis – Experiment B

Angle	emmean	lower.CL	upper.CL
Center	4.843163	3.619890	6.066436
10° right	4.184648	2.742146	5.627150
20° right	3.931448	2.663214	5.199682
30° right	3.264819	2.029927	4.499712
20° top	3.299802	1.684773	4.914832

Code

contrast	estimate	lower.CL	upper.CL	t.ratio	p.value
10° right - Center	-0.6585152	-2.333889	1.0168586	-1.016139	0.7792209
20° right - Center	-0.9117151	-2.317038	0.4936082	-1.679475	0.3427913
30° right - Center	-1.5783437	-2.929355	-0.2273324	-3.024595	0.0159454
20° top - Center	-1.5433610	-3.469405	0.3826833	-2.072063	0.1631420

Table S3 *|Post-hoc comparisons of the SNR values from the different exposure angles in experiment B*

		Prior Odds	Posterior Odds	$BF_{10,U}$	error %
CON	Center	0.260	$9.430 \times 10^{+8}$	$3.628 \times 10^{+9}$	5.939×10^{-15}
	10° horizontal	0.260	$3.860 \times 10^{+7}$	$1.485 \times 10^{+8}$	1.972×10^{-12}
	20° horizontal	0.260	$1.080 \times 10^{+7}$	$4.155 \times 10^{+7}$	7.122×10^{-12}
	30° horizontal	0.260	340870.401	$1.311 \times 10^{+6}$	4.501×10^{-11}
	20° vertical	0.260	409906.587	$1.577 \times 10^{+6}$	4.856×10^{-11}

Table S4 *| Bayesian ANOVA - Post Hoc Comparisons of data from experiment B and the control setting in exp. A.*

Post-hoc analysis: Discomfort

contrast	estimate	SE	df	asymp.LCL	asymp.UCL	z.ratio	p.value
CON - CF	-5.127333	0.1738125	Inf	-5.573862	-4.6808027	-29.499216	0
CON - ISF	-1.906469	0.1817541	Inf	-2.373401	-1.4395368	-10.489277	0
CON - STROBE	-6.248465	0.1562353	Inf	-6.649838	-5.8470914	-39.993949	0
CF - ISF	3.220864	0.1919674	Inf	2.727693	3.7140342	16.778180	0
CF - STROBE	-1.121132	0.1757187	Inf	-1.572559	-0.6697053	-6.380267	0
ISF - STROBE	-4.341996	0.1856599	Inf	-4.818962	-3.8650298	-23.386825	0

Code

contrast	estimate	SE	df	asymp.LCL	asymp.UCL	z.ratio	p.value
Intensity1 - Intensity2	-0.5077064	0.1439768	Inf	-0.8451449	-0.1702679	-3.5263075	0.0012255
Intensity1 - Intensity3	-0.6355818	0.1437226	Inf	-0.9724246	-0.2987391	-4.4222813	0.0000291
Intensity2 - Intensity3	-0.1278754	0.1444997	Inf	-0.4665394	0.2107886	-0.8849529	0.6498193

***Table S5** | Post-hoc comparisons of the discomfort ratings from the different light conditions in experiment C. Note STROBE refers to Luminance Flicker (LF)*

Post-hoc analysis: Flicker

contrast	estimate	SE	df	asymp.LCL	asymp.UCL	z.ratio	p.value
CON - CF	-7.248664	0.1528860	Inf	-7.641433	-6.855895	-47.41220	0
CON - ISF	-3.363502	0.2708508	Inf	-4.059326	-2.667677	-12.41828	0
CON - STROBE	-9.073638	0.1111739	Inf	-9.359247	-8.788028	-81.61663	0
CF - ISF	3.885162	0.1867272	Inf	3.405454	4.364870	20.80662	0
CF - STROBE	-1.824974	0.1043781	Inf	-2.093125	-1.556823	-17.48425	0
ISF - STROBE	-5.710136	0.2043585	Inf	-6.235140	-5.185133	-27.94176	0

Code

contrast	estimate	SE	df	asymp.LCL	asymp.UCL	z.ratio	p.value
Intensity1 - Intensity2	-0.0704570	0.0952025	Inf	-0.2935831	0.1526691	-0.7400753	0.7396176
Intensity1 - Intensity3	-0.0916181	0.0951643	Inf	-0.3146548	0.1314185	-0.9627364	0.6004859
Intensity2 - Intensity3	-0.0211611	0.0937107	Inf	-0.2407910	0.1984687	-0.2258134	0.9722812

***Table S6** | Post-hoc comparisons of the flicker ratings from the different light conditions in experiment C. Note STROBE refers to Luminance Flicker (LF)*

Probability statistics – Experiment C

Condition	Discomfort Rating	Discomfort Probability	Flicker Rating	Flicker Probability
CON 1	0	0.55	0	0.96
CON 2	0	0.43	0	0.96
CON 3	0	0.40	0	0.96
ISF 1	1	0.25	1	0.20
ISF 2	2	0.20	1	0.19
ISF 3	2	0.20	1	0.18
CF 1	6	0.26	7	0.36
CF 2	6	0.25	7	0.35
CF 3	6	0.24	7	0.35
STROBE 1	8	0.25	10	0.39
STROBE 2	8	0.29	10	0.42
STROBE 3	8	0.29	10	0.43

Table S7 *| The 12 light-based stimuli and rating with the highest probability estimated by cumulative linked mixed effects model. Note STROBE refers to Luminance Flicker (LF)*

Supplementary Figures

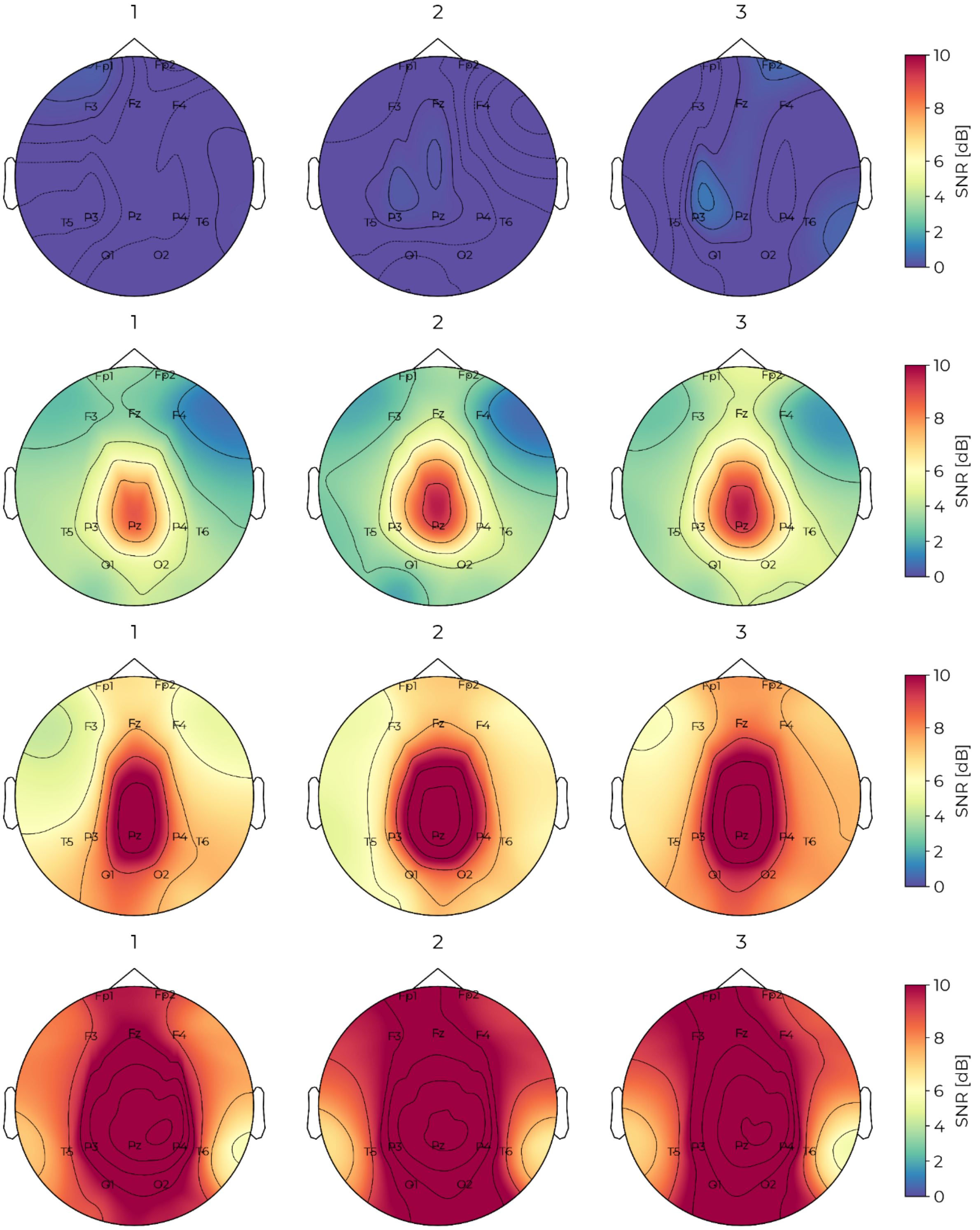

Figure S6 *| Brightness Spatial Response on Common Axis: Row 1: CON, row 2: ISF, row 3: CF, row 4: LF.*

SNR Spatial distribution of brightness levels

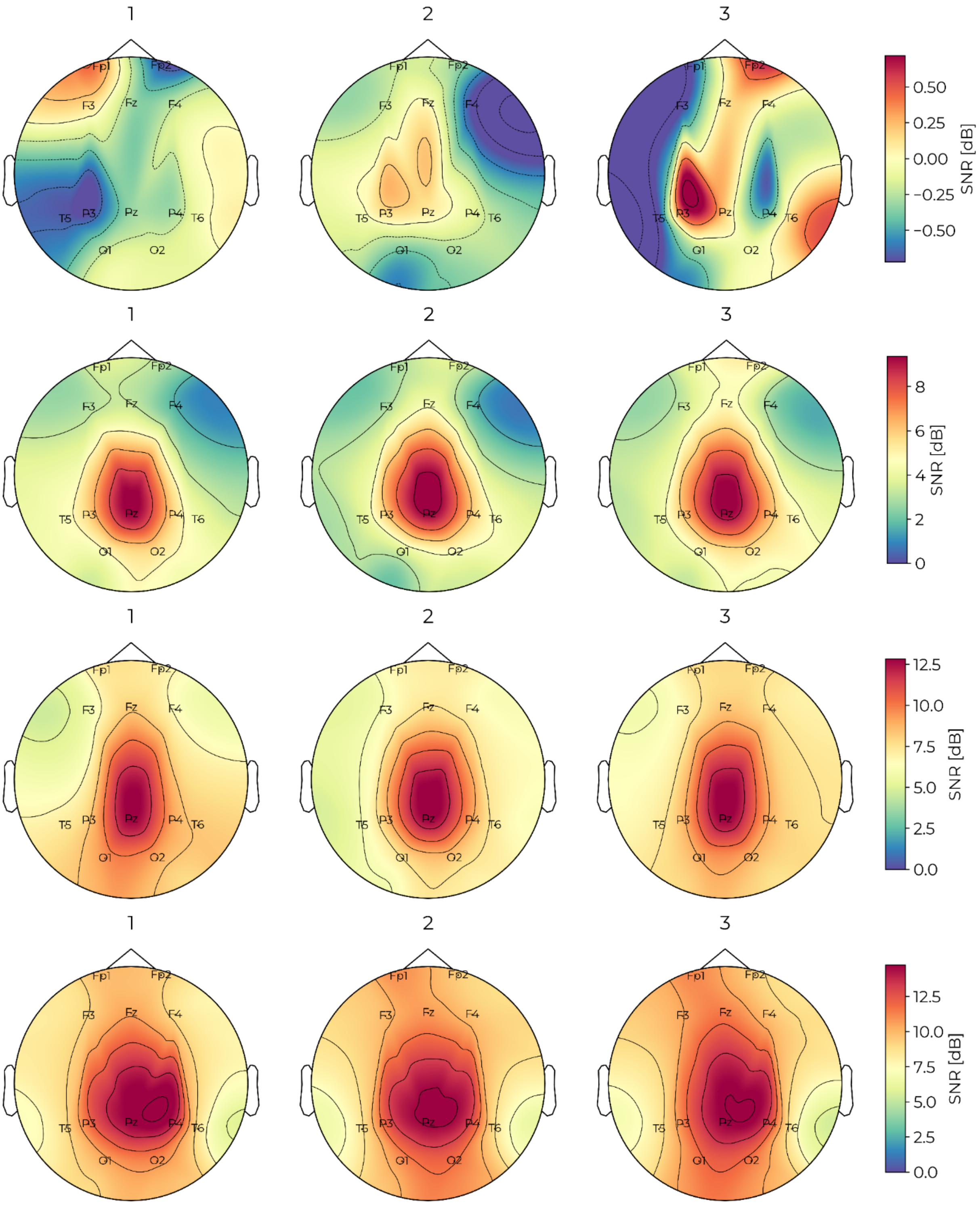

Figure S7 *| Brightness Spatial Response Individual Axis: Row 1: CON, row 2: ISF, row 3: CF, row 4: LF.*
